# Ambient Temperature Preservation Supports Recovery of Beating Capacity in Human Cardiac Organoids After Exposure to Simulated Space Stressors

**DOI:** 10.3390/ijms27198796

**Published:** 2026-09-30

**Authors:** Cynthia Van Rompay, Kevin Tabury, Emre Etlioglu, Ben Cools, Randy Vermeesen, Emil Rehnberg, Sarah Baatout, Marianne S. Carlon, Xavier Casadevall i Solvas, Bjorn Baselet

**Affiliations:** 1 Nuclear Medical Applications, Radiobiology Unit, Belgian Nuclear Research Centre (SCK CEN), 2400 Mol, Belgium; cynthia.van.rompay@sckcen.be or cynthia.vanrompay@kuleuven.be (C.V.R.); emre.etlioglu@sckcen.be (E.E.); ben.cools@sckcen.be (B.C.); randy.vermeesen@sckcen.be (R.V.); emil.rehnberg@ugent.be (E.R.); sarah.baatout@sckcen.be (S.B.); 2 Department of Biosystems, Mechatronics, Biostatistics and Sensors (MeBioS), Biomimetics Group, KU Leuven, 3001 Leuven, Belgium; xevi.casadevall@kuleuven.be; 3 Department of Biomedical Engineering, College of Engineering and Computing, University of South Carolina, Columbia, SC 29208, USA; 4 Department of Pharmaceutical and Pharmacological Sciences, Vrije Universiteit Brussel, 1090 Jette, Belgium; 5 Department of Physics and Astrophysics, Faculty of Sciences, Ghent University, 9000 Ghent, Belgium; 6 Department of Chronic Diseases and Metabolism, Laboratory of Respiratory Diseases and Thoracic Surgery (BREATHE), KU Leuven, 3000 Leuven, Belgium; marianne.carlon@kuleuven.be

**Keywords:** ambient temperature preservation, cardiac organoids, simulated microgravity, ionizing radiation

## Abstract

Human space exploration exposes astronauts to environmental stressors including microgravity (µg) and ionizing radiation, which may adversely affect cardiovascular health. Human induced pluripotent stem cell (hiPSC)-derived cardiac organoids, also referred to as cardioids, provide a physiologically relevant model to investigate these effects, but preservation of complex 3D tissues remains challenging. Current methods rely mainly on cryopreservation, creating variability and logistical constraints, particularly for spaceflight research. Alternative preservation strategies suitable for both ground-based and space experiments are therefore needed. In this study, human cardiac organoids were exposed to simulated µg (sµg) using a random positioning machine (RPM) and to a mixed neutron–photon field generated by a Californium-252 (^252^Cf) source. To assess preservation potential, organoids preserved at ambient temperature in CellShip^®^ transport medium were compared with unpreserved controls. Viability, proliferative activity and functional performance, including spontaneous beating, were assessed. Simulated µg induced limited detectable effects, whereas chronic irradiation (dose equivalent rate, 1.31–1.33 mSv/h over 70.5–74.7 h) impaired several organoid viability and functional readouts. Ambient-temperature-preserved organoids showed greater recovery of measurable beating after irradiation than unpreserved organoids. These proof-of-concept findings support further evaluation of CellShip^®^-based ambient preservation as a non-cryogenic alternative for cardiac organoids in terrestrial and spaceflight research.

## 1. Introduction

Human space exploration exposes astronauts to a unique combination of environmental stressors, including µg and ionizing radiation, which together can have profound effects on physiological systems. Among these, the cardiovascular system is particularly susceptible, with reported alterations in cardiac structure, function, and cellular homeostasis during and after spaceflight [1,2,3,4]. Understanding the underlying mechanisms of these changes is essential for assessing health risks and developing countermeasures for long-duration missions, such as those planned for deep-space exploration.

To investigate these effects, both spaceflight experiments conducted onboard platforms such as the International Space Station (ISS) and ground-based analog systems are widely used. Although experiments aboard the ISS provide direct access to the spaceflight environment, they remain constrained by cost, access, and logistical constraints, including pre-launch sample preparation and preservation requirements. Ground-based simulation platforms are essential tools for investigating the biological effects of microgravity in a controlled and accessible manner. Among these, the random positioning machine (RPM) is widely used to mimic aspects of microgravity by continuously randomizing the orientation of the gravitational vector through independent rotation around two perpendicular axes, thereby canceling out the perceived gravitational force over time. Successful simulation of microgravity requires rotation frequencies that exceed the timescale of gravity-sensitive cellular processes while minimizing centrifugal forces [5,6,7]. Neutron-emitting sources such as ^252^Cf generate mixed neutron–photon fields with high relative biological effectiveness (RBE) and can be used to investigate responses to secondary neutron components relevant to shielded space environments, but they do not reproduce the full energy, particle, or dose-rate spectrum of galactic cosmic radiation (GCR) [8,9]. Chronic ^252^Cf exposure can deliver a dose equivalent of approximately 100 mSv within an experimentally practical interval. This cumulative value is comparable in magnitude to reported exposures during a six-month stay on the ISS, although the radiation quality and dose rate differ substantially [10]. Investigating organ-specific responses to space-related stressors requires advanced experimental platforms, such as human 3D in vitro models, including organoids derived from hiPSCs. 3D cardiac models can recapitulate key aspects of myocardial structure and function, including spontaneous contractility, making them highly relevant for investigating cardiovascular responses to (simulated) µg and radiation [11,12,13,14,15,16].

A critical challenge in both terrestrial and space-based experiments is the preservation of complex 3D cellular models prior to and during exposure to experimental conditions to maintain viability and functional properties for long-duration experiments. Current approaches predominantly rely on cryopreservation, which requires specialized infrastructure that increases logistical complexity. Moreover, variability in post-thaw cell viability as well as function can arise due to factors such as cryoprotective agent (CPA) toxicity, osmotic stress, and sensitivity to cooling and warming rates, all of which critically influence post-thaw cell survival [17]. Post-thaw variability in cell viability and function has been reported for cardiac cells, including impairments in contractility, electrophysiological properties, and metabolic activity, largely due to CPA toxicity, osmotic stress, and altered cooling and warming rates [18]. These human iPSC-derived cardiac 3D models have already been implemented in spaceflight studies; in this context, cryopreserved 3D hiPSC-derived cardiac progenitor spheroids have been successfully transported to the ISS, thawed in orbit, and cultured to yield viable and functionally active cardiomyocytes [14]. Furthermore, earlier work has demonstrated that spaceflight can induce changes in cardiomyocyte calcium handling and mitochondrial function, highlighting the sensitivity of cardiac cells to the space environment [19]. Alternative preservation strategies, particularly those based on ambient temperature conditions, could simplify workflows, reduce resource requirements and enhance flexibility in experimental design for terrestrial and spaceflight applications. However, the impact of such preservation methods on the viability and function of advanced 3D models, especially under combined space-related stressors, remains poorly understood.

In this study, we investigated the effects of simulated space conditions on human cardiac organoids, cardioids, focusing on the combined and individual impact of simulated µg (sµg) through application of the RPM and chronic neutron radiation by a ^252^Cf source. Importantly, we compared unpreserved organoids with organoids preserved at ambient temperature using CellShip^®^ transport medium (Life Science Production, SCS-004K, Bedfordshire, UK) while exposed, in order to evaluate whether this preservation strategy influences cellular responses to space-related stressors. By integrating advanced 3D cardiac models with both environmental simulation and preservation approaches, this work aims to contribute to the evaluation of more accessible and scalable methods for biological space research, while also improving future experimental strategies for studies conducted both on Earth as well as in spaceflight environments such as the ISS and beyond.

## 2. Results

**Figure 1:**
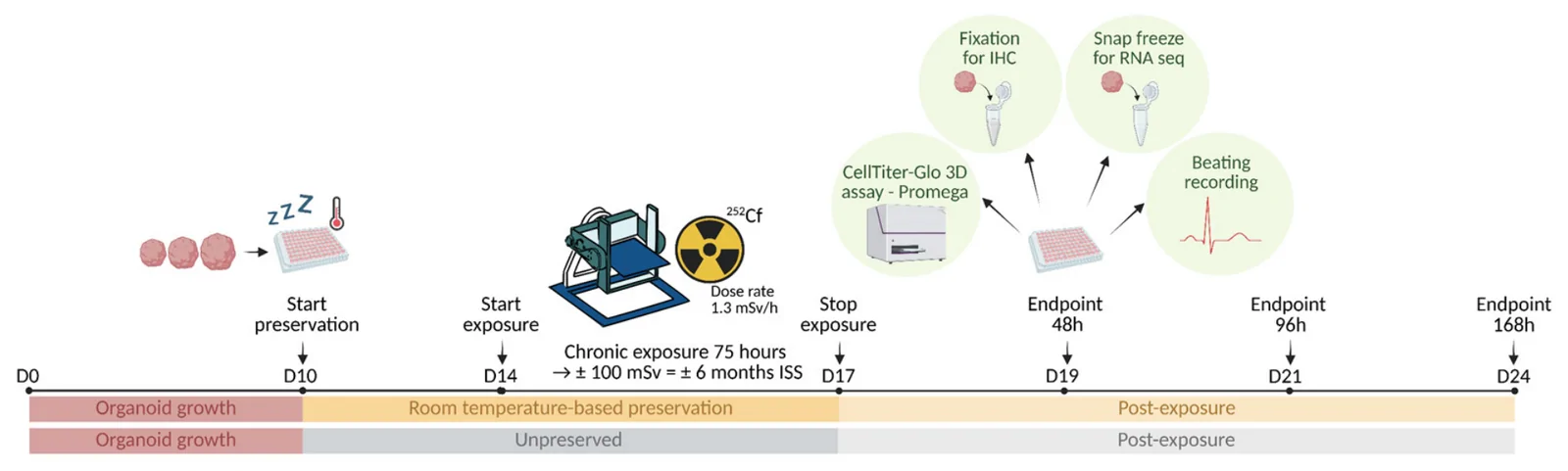
Overview of our study design. D: day; h: hours; ^252^Cf: Californium-252; mSv/h: millisievert per hour. ISS: International Space Station; IHC: immunohistochemistry; RNA seq: RNA extraction with subsequent sequencing. Created in BioRender. Bijnens, M. (2026) https://BioRender.com/fisi4qn.

Ten-day-old cardiac organoids were generated, cultured and preserved at ambient temperature as previously described [13,20]. After four days of preservation, in the middle of the total seven-day preservation period, exposure to simulated space stressors was started. The RPM was used to simulate µg and a ^252^Cf source was applied at an average dose equivalent rate of 1.3 mSv/hour to irradiate the samples with high-linear energy transfer (LET) radiation as one of the components of the GCR spectrum. Both stressors were applied individually and samples were simultaneously exposed as well. Samples received a cumulative dose of approximately 92.5–99.5 mSv, approaching the 100 mSv corresponding to a six-month stay on the ISS (Figure 1) [10]. Preservation of cardiac organoids was halted after exposure to simulated space stressors to verify recovery capacity upon exposure. Unpreserved controls were maintained under standard culture conditions in a humidified incubator and directly subjected to the subsequent experimental exposures without undergoing preservation.

### 2.1. Simulated Microgravity Produces Limited Effects on Cardiac Organoid Viability and Function Under the Conditions Tested

**Figure 2:**
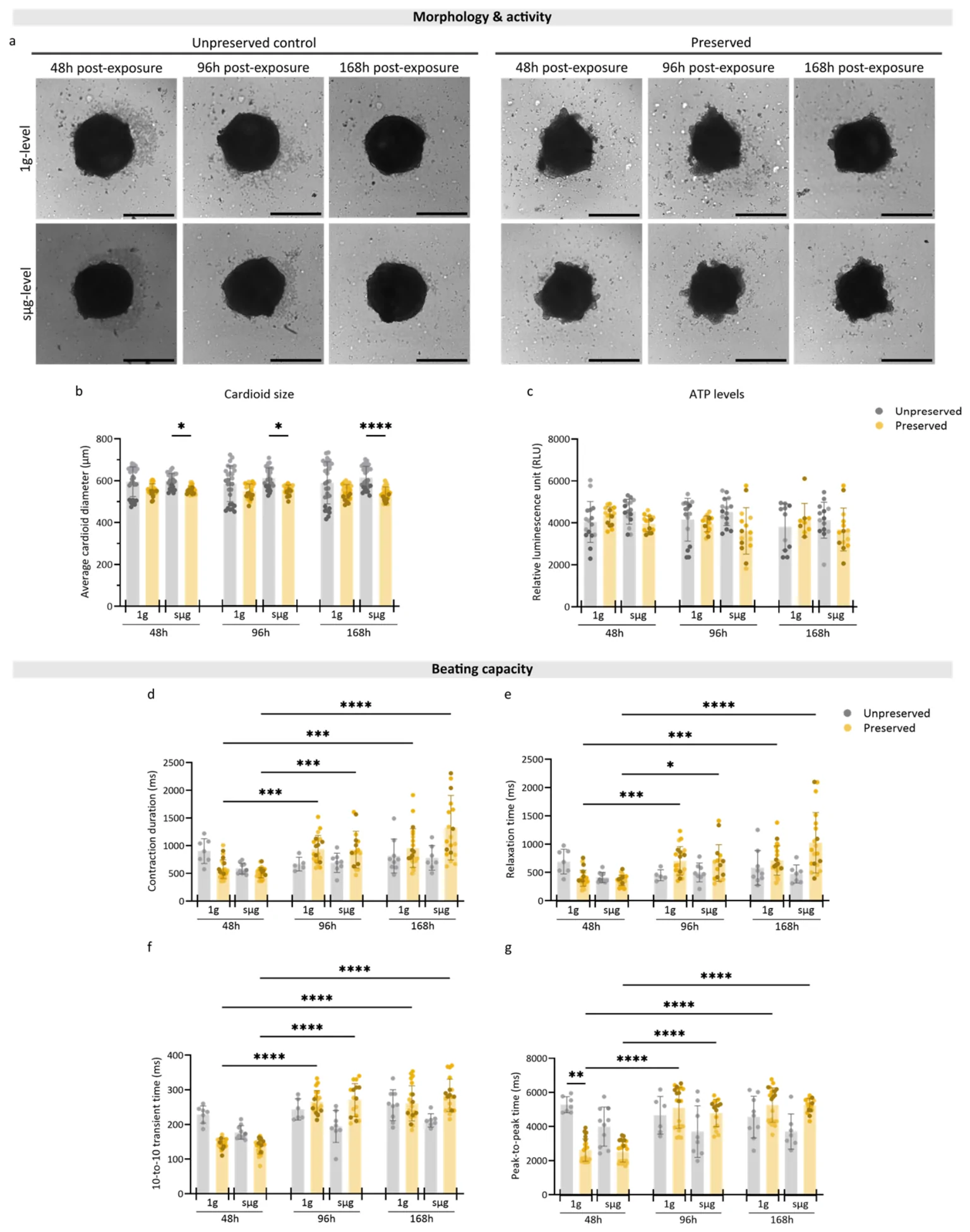
Simulated microgravity (sµg) does not affect size, ATP levels and beating capacity of cardiac organoids. (**a**) Brightfield images of unpreserved and preserved cardiac cardioids post-exposure to sµg-level and 1g-level. Scale bar: 500 µm. (**b**) Average cardioid diameter upon exposure to simulated µg. (**c**) Averaged cardioid ATP levels post-exposure. ATP: adenosine triphosphate. Beating parameters of cardiac organoids: contraction duration (**d**), relaxation time (**e**), 10-to-10 transient time (**f**) and peak-to-peak time (**g**) in milliseconds (ms). Shades represent biological replicates (N = 3). The data are presented as the means ± SD. The Kruskal–Wallis test with Dunn’s multiple comparisons test was used. * *p* < 0.05; ** *p* < 0.01; *** *p* < 0.001; **** *p* < 0.0001.

Preserved and unpreserved cardiac organoids were exposed to simulated µg levels to evaluate the effect of this space-related stressor. Several readouts were implemented to evaluate organoid health under simulated µg levels, namely via beating capacity, viability, size, ATP measurements, proliferation capacity and degree of apoptosis. Based on brightfield images of unpreserved controls, no significant difference in organoid diameter was detected over time post-exposure (Figure 2a,b). However, preserved organoids exposed to simulated µg had a significant smaller diameter compared to unpreserved controls at the 48 h, 96 h and 168 h post-exposure timepoints, with mean diameter of 555 ± 19 µm (*p* < 0.05), 558 ± 22 µm (*p* < 0.05), and 539 ± 31 µm, respectively (Figure 2a,b). Despite this reduction in size, ATP-level detection did not show a corresponding decrease in metabolic activity (Figure 2c), suggesting that the small difference in organoid size did not affect ATP levels. Visual observation suggested that more unpreserved cardiac organoids continued beating upon exposure to simulated µg, although the opposite was observed in preserved organoids (Supplementary Materials—Figure S1a). Analysis of this beating capacity in preserved organoids demonstrated an extended contraction duration, relaxation time, 10-to-10 transient time and peak-to-peak time at 96 h and 168 h post-exposure, as well as for unexposed preserved organoids compared to an earlier 48 h timepoint (Figure 2d–g). While this effect was present in preserved organoids, it was not detectable for unpreserved organoids over time or when comparing to control the 1g-level at each individual timepoint. In addition, at 48 h post-exposure preserved organoids at the 1g level were beating with a higher frequency compared to unpreserved controls as evidenced by a reduced peak-to-peak time (Figure 2g).

**Figure 3:**
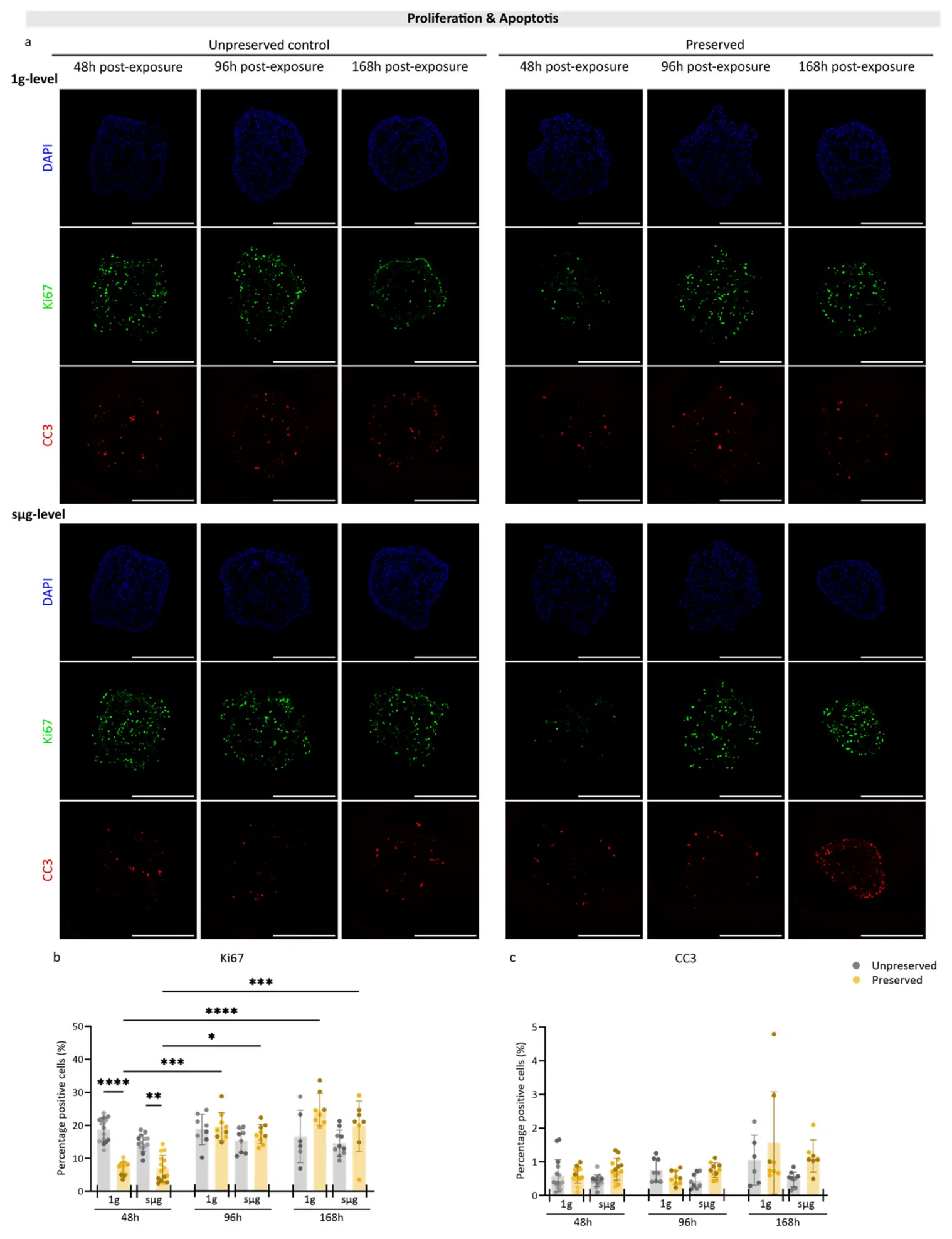
Proliferation capacity and apoptosis in cardiac organoids is not affected by simulated microgravity (sµg) exposure. (**a**) Fluorescence images of cardiac organoid cross sections. Scale bar: 300 µm. DAPI: nuclear marker; Ki67: proliferation marker; CC3: apoptosis marker. (**b**) The percentage of Ki67-positive cells, which is indicative of the number of proliferative cells (N = 3). (**c**) The percentage of CC3-positive cells, which is indicative of the number of apoptotic cells (N = 3). The data are presented as mean ± SD, and the Kruskal–Wallis test with Dunn’s multiple comparisons test was used. * *p* < 0.05; ** *p* < 0.01; *** *p* < 0.001; **** *p* < 0.0001.

Furthermore, immunofluorescence images of cardioid cross sections revealed an average of 7% Ki67-positive cells at 48 h post-exposure for preserved organoids at the 1g-level and simulated µg-level, which increased slightly over time, with 20% (1g) and 17% (sµg) at 96 h and 25% (1g) and 20% (sµg) at 168 h post-exposure, and after preservation (Figure 3a,b). Additionally, less Ki67-positive cells were detected in preserved organoids (7% Ki67-positive cells) compared to unpreserved controls (19% Ki67-positive cells), both at normal 1g-levels (*p* < 0.0001) and sµg-levels (*p* < 0.01) after 48 h. Moreover, no significant differences were observed in CC3-positive cells.

Overall, no statistically significant changes attributable to 75 h of RPM exposure were detected in the selected viability and functional readouts for unpreserved cardioids. This absence of detected differences should not be interpreted as evidence that simulated µg has no biological effect, particularly given the exposure duration and mechanical constraints of the RPM configuration. However, comparison of preserved and unpreserved organoids at both gravity levels revealed transient changes in beating and proliferation capacity at 48 h, including fewer Ki67-positive cells, with values approaching control levels at later timepoints. A modest reduction in diameter persisted in preserved organoid exposed to simulated µg.

### 2.2. ^252^Cf Radiation Alters Viability and Beating Function of Cardiac Organoids

**Figure 4:**
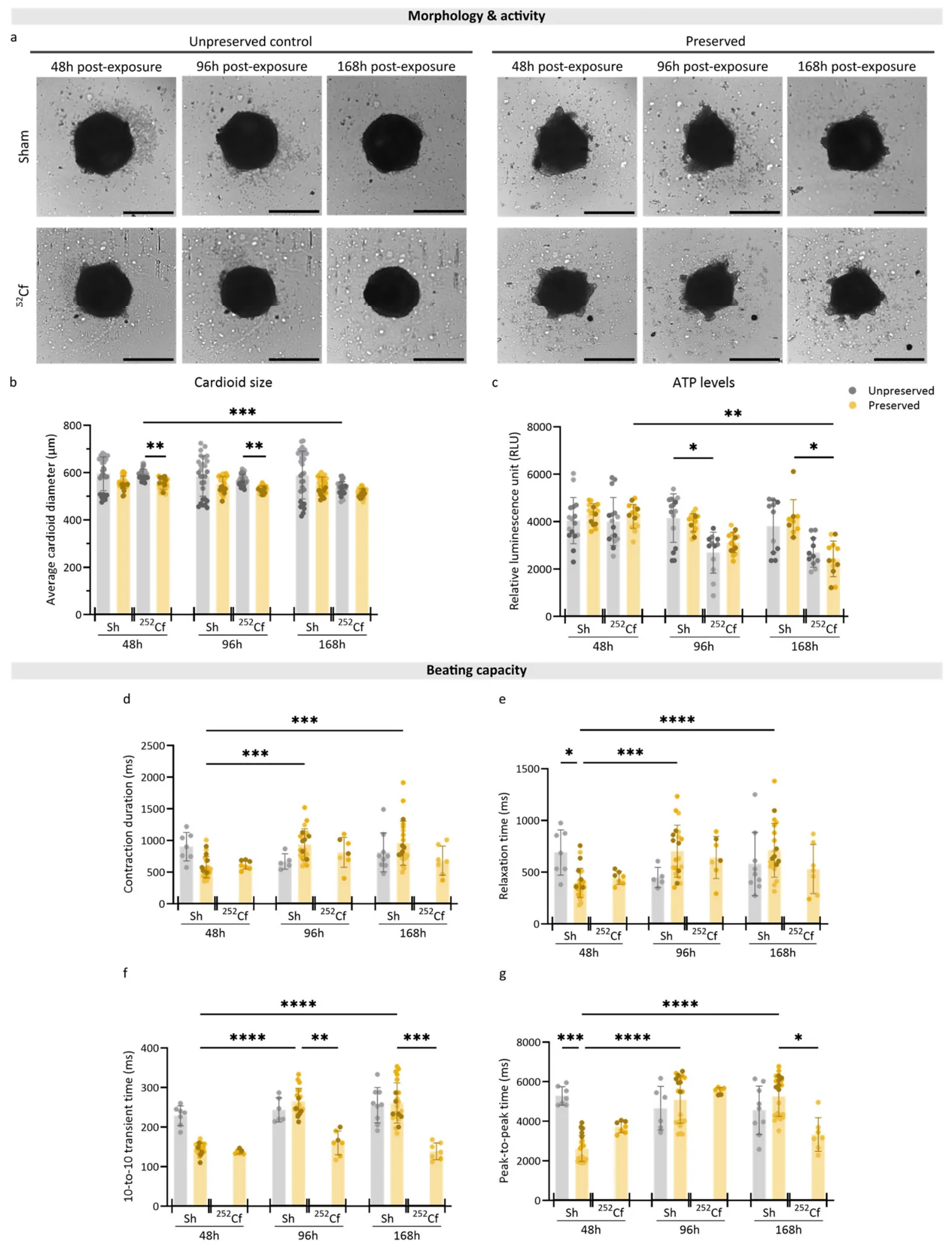
^252^Cf radiation reduces size, ATP levels and beating capacity of cardiac organoids. (**a**) Brightfield images of unpreserved and preserved cardiac cardioids post-exposure to ^252^Cf radiation. Scale bar: 500 µm. (**b**) Average cardioid diameter of sham and irradiation cardiac organoids. (**c**) Averaged cardioid ATP levels of sham and irradiated cardiac organoids. ATP: adenosine triphosphate. Beating parameters of cardiac organoids: contraction duration (**d**), relaxation time (**e**), 10-to-10 transient time (**f**) and peak-to-peak time (**g**) in milliseconds (ms). Shades represent biological replicates (N = 3). The data are presented as the means ± SD. The Kruskal–Wallis test with Dunn’s multiple comparisons test was used. * *p* < 0.05; ** *p* < 0.01; *** *p* < 0.001; **** *p* < 0.0001.

Irradiation with a ^252^Cf neutron source was used to evaluate the effect of chronic neutron exposure on the viability and function of preserved and unpreserved cardiac organoids. Size measurements based on brightfield images (Figure 4a) showed a slightly reduced organoid diameter of irradiated samples after 48 h and 96 h post-exposure while preserved, i.e., an average diameter of 552 ± 23 µm after 48 h; 531 ± 14 µm (*p* < 0.05) after 96 h, unpreserved irradiated samples had an average diameter of 593 ± 22 µm after 48 h and 570 ± 23 µm after 96 h (Figure 4b). However, this effect was not detected at the final timepoint, after 168 h. When evaluating the effect of radiation individually by measuring the size of unpreserved organoids over time post-irradiation, a significantly reduced diameter was observed at 168 h (534 ± 27 µm) compared to 48 h (593 ± 22 µm) (*p* < 0.001) (Figure 4b). Evaluation of ATP levels of irradiated, unpreserved organoids revealed a significantly reduced ATP level of 35% at 96 h post-exposure compared to sham controls (*p* < 0.05) (Figure 4c). Irradiation of preserved cardioids induced a significant ATP reduction of 41% at 168 h compared to sham (*p* < 0.05) and a reduction of 43% over time when comparing ATP levels at 168 h to levels at 48 h post-exposure (*p* < 0.01) (Figure 4c). These results are indicative of an adverse radiation effect on cardiac organoid ATP levels, occurring earlier in time post-irradiation when unpreserved compared to preserved samples. Moreover, this positive effect of preservation while being irradiated was highlighted when observing beating capacity post-exposure. Here, a decreasing trend in number of beating organoids over time was detected when organoids were unpreserved, while the trend was reversed in organoids that were preserved, resulting in more beating organoids when they were preserved at ambient temperature (Supplementary Materials—Figure S1b). Moreover, beating capacity of unpreserved irradiation samples was not measurable by the MUSCLEMOTION tool, represented by the absence of data points for this condition in the bar plots (Figure 4d–g). Interestingly, organoids that were preserved while chronically exposed to ^252^Cf irradiation retained beating capacity post-irradiation, which highlights a beneficial effect of the preservation methods when exposed to this type of radiation. Analysis of beating capacity of preserved sham organoids showed a significant increasing trend at 96 h and 168 h compared to 48 h post-exposure for several parameters including contraction duration, relaxation time, 10-to-10 transient time and peak-to-peak time (Figure 4d–g). This is indicative of longer and less frequent contractions that are held longer in the contracted state. At 48 h post-exposure, sham preserved organoids differed from unpreserved organoids in relaxation time and peak-to-peak time, with lower levels detected due to preservation (Figure 4e,g). The effect of radiation on preserved cardiac organoids was highlighted by a decreased 10-to-10 transient time at 96 h and 168 h for preserved irradiated organoids, as well as a decreased peak-to-peak time at 168 h post-exposure for the latter condition (Figure 4f,g). This is indicative of radiation increasing the beating frequency and reducing the time held in the contracted state for preserved cardiac organoids.

**Figure 5:**
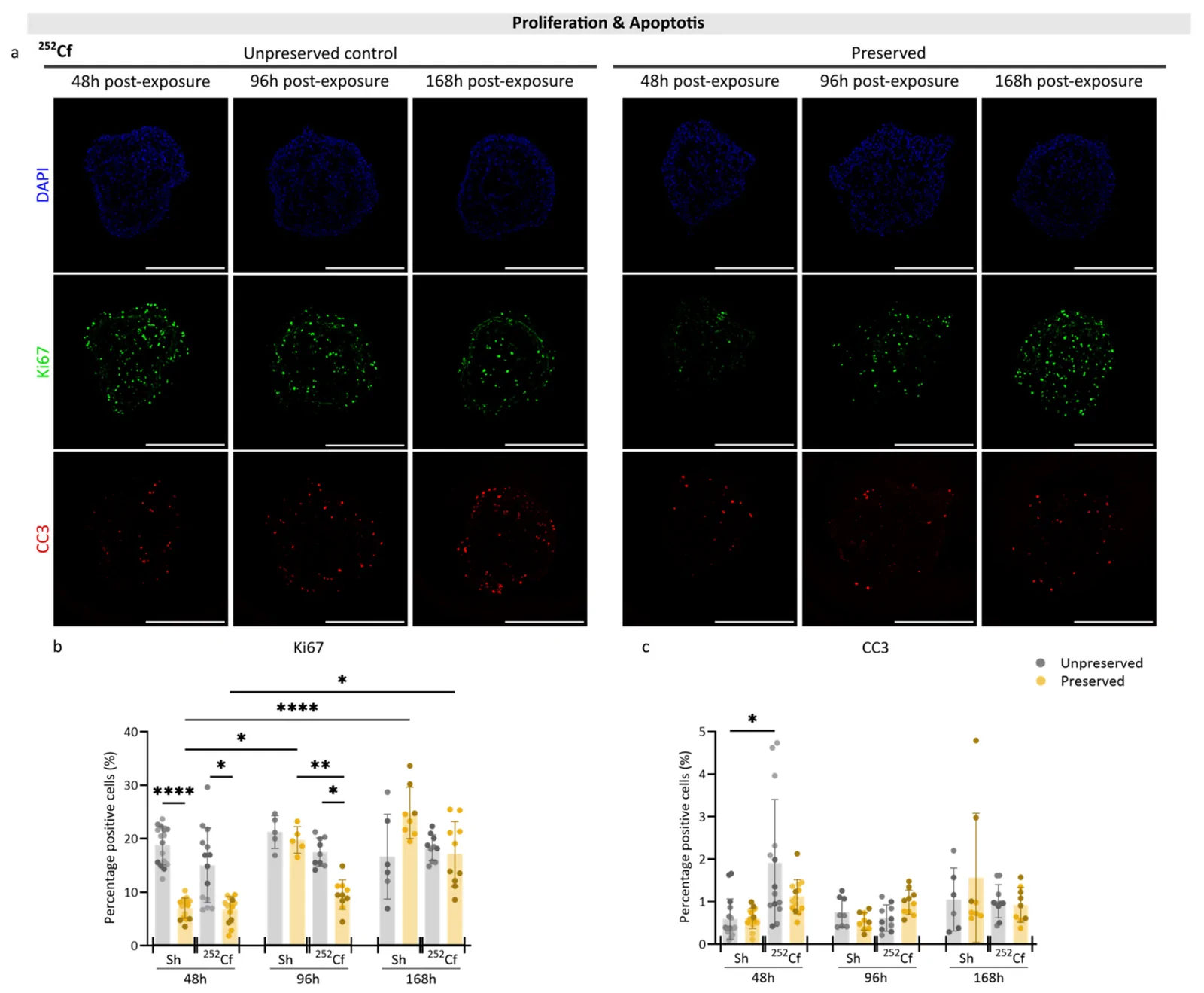
Exposure to ^252^Cf radiation initially decreases proliferation capacity in preserved cardiac organoids and induces apoptosis in unpreserved cardiac organoids. (**a**) Fluorescence images of cardiac organoid cross sections. Scale bar: 300 µm. DAPI: nuclear marker; Ki67: proliferation marker; CC3: apoptosis marker. (**b**) The percentage of Ki67-positive cells, which is indicative of the number of proliferative cells (N = 3). (**c**) The percentage of CC3-positive cells, which is indicative of the number of apoptotic cells (N = 3). The data are presented as mean ± SD, and the Kruskal–Wallis test with Dunn’s multiple comparisons test was used. * *p* < 0.05; ** *p* < 0.01; **** *p* < 0.0001.

Immunofluorescence images of cardioid cross sections revealed an average of 7% Ki67-positive cells at 48 h post-exposure for preserved sham organoids, which increased slightly over time, with 20% Ki67-positive cells at 96 h (*p* < 0.05) and 25% at 168 h post-exposure (*p* < 0.0001) (Figure 5a,b). Additionally, an increasing trend was detected over time for irradiated preserved organoids, with 7% Ki67-positive cells at 48 h and 17% Ki67-positive cells at 168 h (*p* < 0.0001) (Figure 5a,b). Less Ki67-positive cells were detected in preserved sham organoids (7% Ki67-positive cells) compared to unpreserved sham controls (19% Ki67-positive cells) (*p* < 0.0001) (Figure 5a,b). This adverse preservation effect was also present in irradiated organoids after 48 h and 96 h post-exposure, where 15% (48 h) and 18% (96 h) Ki67-positive cells were detected in unpreserved organoids and only 7% (48 h) and 10% (96 h) Ki67-positive cells in preserved organoids (*p* < 0.05) (Figure 5a,b). Interestingly, ^252^Cf radiation decreased proliferation capacity in preserved organoids (10% Ki67-positive cells) compared to sham at 96 h post-exposure (20% Ki67-positive cells) (*p* < 0.01) (Figure 5a,b). Moreover, a significant difference in CC3-positive cells was observed when comparing unpreserved irradiated (2% CC3-positive cells) organoids with sham controls (0.6% CC3-positive cells) at 48 h post-exposure (*p* < 0.05) (Figure 5a,c). Despite the variation between replicates, these results could indicate an initial response to radiation exposure, resulting in more apoptosis when unpreserved and less proliferative cells when preserved, but these deviations recover to levels similar to controls over time after exposure.

Overall, ^252^Cf irradiation altered ATP-related and contractile readouts in this cardioid model and was associated with an early increase in apoptosis in unpreserved organoids and reduced proliferation at 96 h in preserved organoids. Measurable beating was absent in unpreserved irradiated samples but remained detectable after recovery in preserved irradiated organoids. Because preservation simultaneously changes temperature, medium composition, and metabolic state during exposure, these findings support an association between the preservation condition and post-irradiation recovery of beating rather than demonstrating radioprotection per se.

### 2.3. Effects Observed Following Combined Exposure to ^252^Cf Radiation and Simulated Microgravity

**Figure 6:**
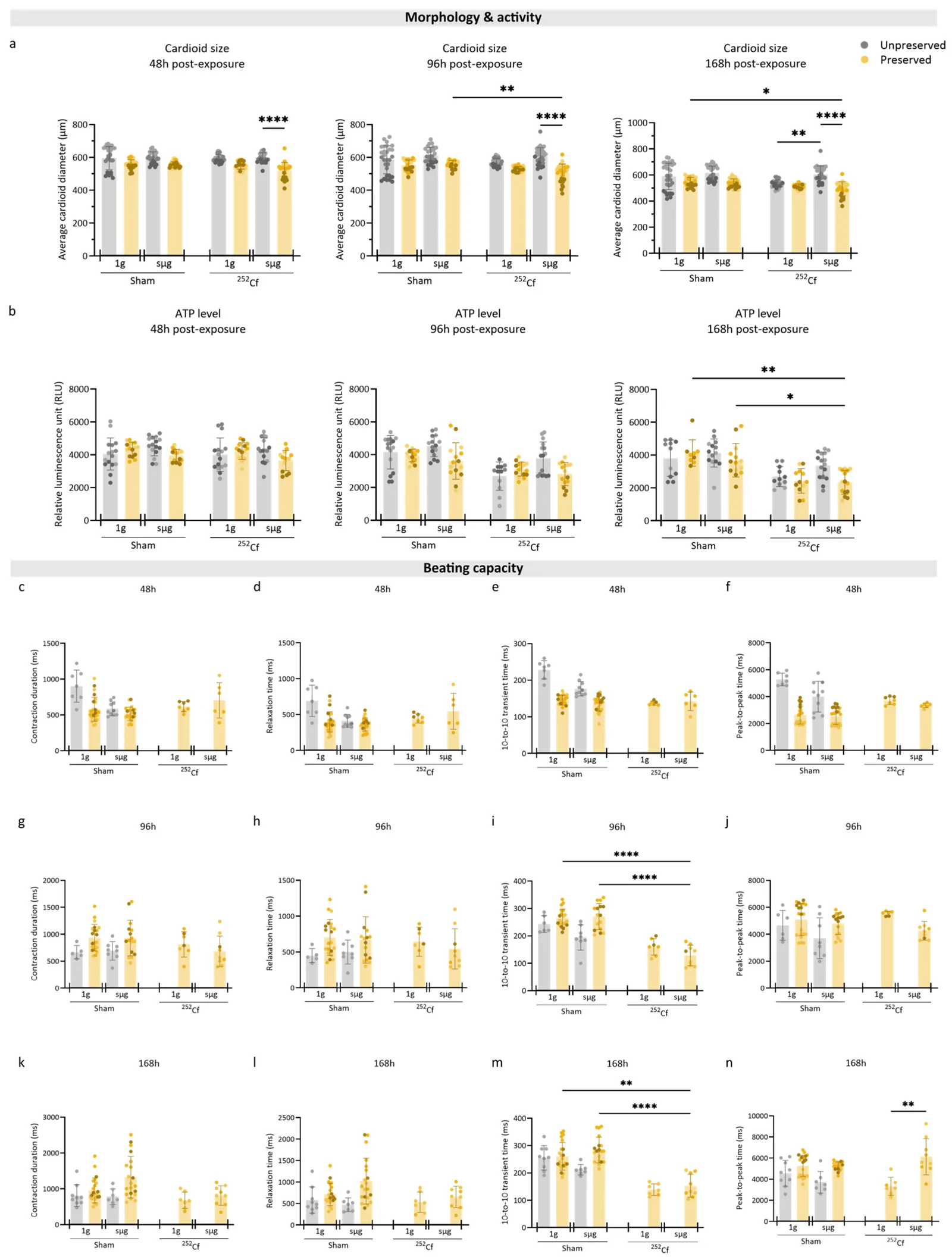
Combined exposure decreases size and ATP levels of preserved cardiac organoids, while retaining beating capacity. (**a**) Average cardioid diameter of sham and irradiation cardiac organoids after 48 h, 96 h and 168 h post-exposure. (**b**) Averaged cardioid ATP levels of sham and irradiated cardiac organoids after 48 h, 96 h and 168 h post-exposure. ATP: adenosine triphosphate. Beating parameters of cardiac organoids after 48 h, 96 h and 168 h post-exposure: contraction duration (**c**,**g**,**k**), relaxation time (**d**,**h**,**l**), 10-to-10 transient time (**e**,**i**,**m**) and peak-to-peak time (**f**,**j**,**n**) in milliseconds (ms). Shades represent biological replicates (N = 3). The data are presented as the means ± SD, and the Kruskal–Wallis test with Dunn’s multiple comparisons test was used. * *p* < 0.05; ** *p* < 0.01; **** *p* < 0.0001.

Combined exposure of cardiac organoids to ^252^Cf radiation and simulated µg allows the investigation of possible antagonistic or amplified effects of both simulated space stressors. Previously discussed individual exposure and/or preservation effects (represented in Figure 2, Figure 3, Figure 4 and Figure 5) were not indicated here to highlight the effects of combined exposure to ^252^Cf radiation and simulated µg. Cardiac organoids size measurements showed a decreased average diameter for preserved organoids when exposed to simulated µg-levels and radiation at all timepoints compared to unpreserved controls (*p* < 0.0001) (Figure 6a; Supplementary Materials—Figure S2). At 168 h post exposure, the average diameter was reduced by 12% for unpreserved organoids (534 ± 27 µm) that were solely exposed to irradiation compared to exposure to combined stressors (605 ± 65 µm) (*p* < 0.01) (Figure 6a), indicative of a beneficial simulated µg effect while irradiated. Additionally, average cardioid diameter was reduced with 9% at 96 h post-exposure for preserved ^252^Cf-irradiated sµg-level samples (508 ± 51 µm) compared to preserved sham sµg-level samples (558 ± 22 µm) (*p* < 0.01), indicative of an adverse radiation effect added to simulated µg exposure (Figure 6a). This effect was also observed when evaluating ATP levels at 168 h post-exposure where an average decrease of 37% in ATP levels was detected when preserved and exposed to combined stressors compared to sham sµg-levels (*p* < 0.05) (Figure 6b). Interestingly, at the 168 h timepoint, combined exposure reduced the average organoid diameter by 8% (*p* < 0.05) and the ATP levels by 41% in preserved samples compared to sham controls at the 1g-level (*p* < 0.01) (Figure 6a,b). This effect of combined exposure was not observed in unpreserved cardiac organoids, which could imply higher sensitivity of cardiac organoids to both space stressors when preserved. However, when comparing preserved versus unpreserved size and ATP levels when irradiated and exposed to sµg-levels, significant differences were only found in size, not in ATP levels.

Assessment of beating capacity showed that more organoids were beating when preserved and exposed to combined space stressors compared to unpreserved controls (Supplementary Materials—Figure S1c). Additionally, the absence of measurable beating capacity in unpreserved organoids that were irradiated or subjected to combined exposure implies a protective effect of the preservation method on the beating capacity of our cardiac organoid model post-exposure (Figure 6c–n). Furthermore, no significant differences in beating capacity compared to controls were observed at the earliest timepoint of 48 h post-exposure. At 96 h and 168 h post-exposure, ^252^Cf-radiation in combination with simulated µg-level exposure reduced the 10-to-10 transient time, i.e., the time in contracted state, in preserved cardiac organoids compared to the 1g-level and sµg-level controls (*p* < 0.0001) (Figure 6i,m). This effect was also observed in preserved cardiac organoids that were irradiated, without simulated µg exposure (Figure 4e), which could imply a radiation effect rather than an amplified effect of combined exposures. Furthermore, an increased peak-to-peak time was detected in preserved cardiac organoids at the sµg-level in combined exposure when comparing to 1g-level at 168 h post-exposure (*p* < 0.01) (Figure 6n), which can be indicative of a sµg effect in irradiated samples.

**Figure 7:**
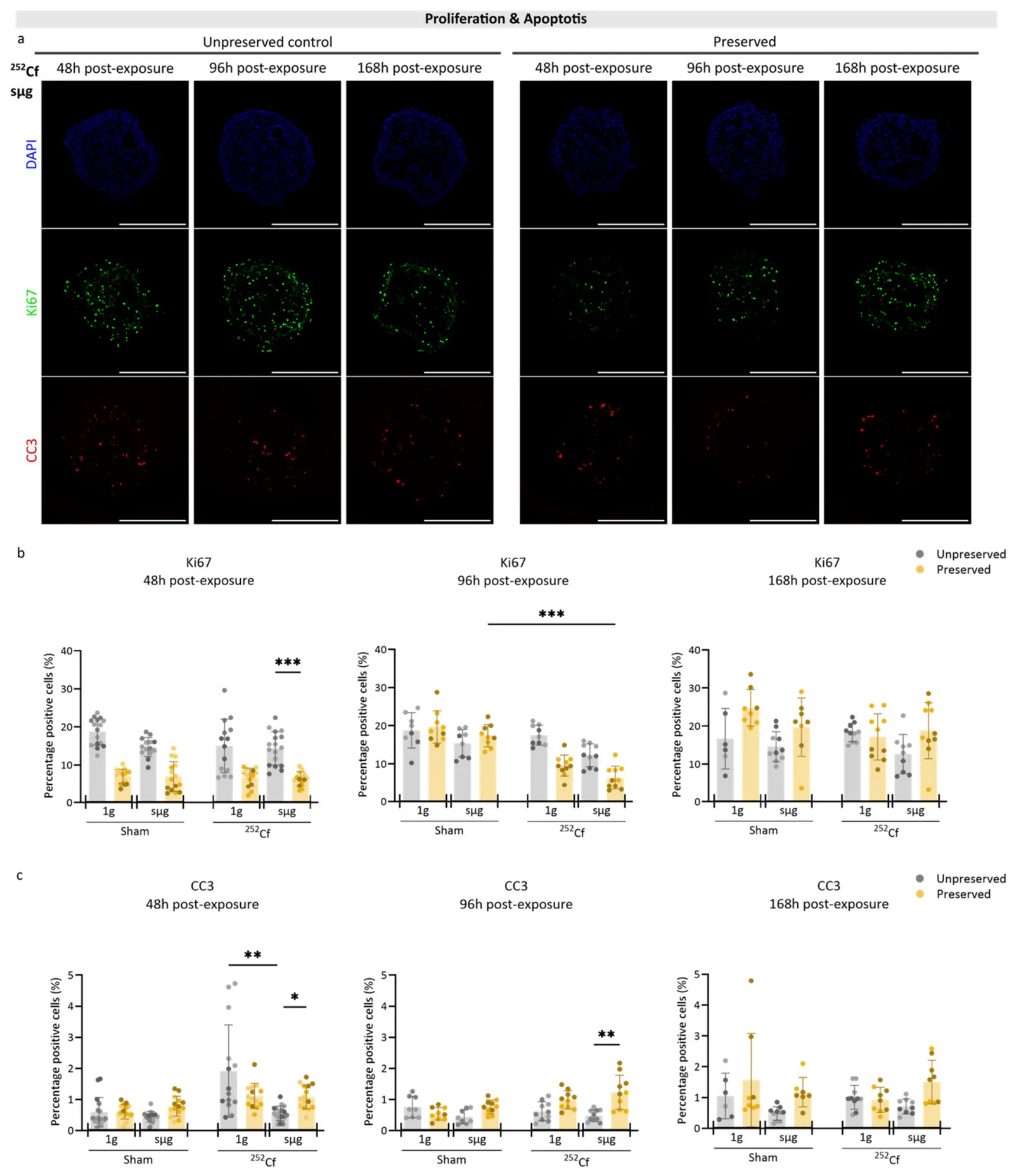
Combined exposure of space stressors does not affect proliferation and apoptosis levels of cardiac organoids more than individual exposures. (**a**) Fluorescence images of cardiac organoid cross sections. Scale bar: 300 µm. DAPI: nuclear marker; Ki67: proliferation marker; CC3: apoptosis marker. (**b**) The percentage of Ki67-positive cells, which is indicative of the number of proliferative cells (N = 3). (**c**) The percentage of CC3-positive cells, which is indicative of the number of apoptotic cells (N = 3). The data are presented as mean ± SD, and the Kruskal–Wallis test with Dunn’s multiple comparisons test was used. * *p* < 0.05; ** *p* < 0.01, *** *p* < 0.001.

Furthermore, immunofluorescence images of cardioid cross sections showed no additional differences in proliferation capacity and apoptosis when exposed to both simulated space stressors in unpreserved cardioids compared to controls (Figure 7). The combination of ^252^Cf-radiation and simulated µg while preserved reduced the number of Ki67-positive cells by 56% after 48 h post-exposure (*p* < 0.001) and increased apoptosis by 49% and 61% after 48 h and 96 h, respectively, compared to unpreserved controls (Figure 7a–c). This adverse preservation effect was restored to control levels, with enhanced proliferation in preserved organoids after 96 h and less apoptosis after 168 h (Figure 7a,b). Furthermore, 63% less Ki67-positive cells were observed when comparing combined exposure to sµg-level exposure in preserved cardiac organoids after 96 h (*p* < 0.001) (Figure 7a,b), which could imply an adverse radiation effect at this timepoint, affecting the rate of regaining proliferative capacity. It is of interest that this effect was not observed when evaluating cell death post-exposure (Figure 7a,c). Here, combined exposure in unpreserved organoids resulted in 71% less CC3-positive cells compared to 1g-level irradiated organoids (*p* < 0.01), an effect that was present after 48 h, probably due to high variation between replicates in the latter condition since this effect was absent in later timepoints (Figure 7a,c).

Overall, combined exposure was associated with reduced size, ATP levels and 10-to-10 transient time in preserved cardiac organoids at 168 h post-exposure. Preserved cardiac organoids retained measurable beating after combined exposure to ^252^Cf radiation and simulated µg, whereas beating could not be quantified in the corresponding unpreserved conditions. However, most observed alterations were also present after individual exposure or preservation.

### 2.4. Ambient Preservation-Induced Alterations upon Exposure to Simulated Space Stressors Normalize over Time Without Loss of Beating Function

In addition to the evaluation of the effects of simulated space stressors, both individually and combined, for preserved and unpreserved cardiac organoids, the effects of the preservation itself were considered. Comparison of preserved to unpreserved cardiac organoids, irrespective of g-levels, revealed an initial adverse effect of preservation on beating and proliferation capacity at 48 h via reduced number of Ki67-positive cells, while this effect was restored to control levels at later timepoints (Figure 2 and Figure 3). More specifically for preserved organoids only exposed to simulated µg-levels, reduced size measurements were observed at all timepoints due to an adverse preservation effect occurring when exposed to simulated µg-levels (Figure 2 and Figure 3). This implies that some adverse preservation effects, regarding size, proliferation and beating capacity, are present 48 h post-exposure, but more effective when exposed to simulated µg-levels. Interestingly, these alterations are restored to levels similar to unpreserved controls over time after exposure, except for the reduced cardioid size.

Furthermore, ATP levels and beating capacity of cardiac organoids were affected by neutron irradiation. Additionally, an initial increase in apoptosis at 48 h post-exposure in unpreserved organoids and a decreased proliferation capacity in preserved organoids after 96 h (Figure 4 and Figure 5) were noted. However, these effects restored to control levels over time, indicating that preserved organoids initially needed more time to regain proliferative capacity. Interestingly, comparison of preserved with unpreserved irradiated organoids revealed the absence of measurable beating capacity when unpreserved, while preserved organoids retained their beating capacity post-exposure (Figure 4 and Figure 5). These findings could imply a possible beneficial effect of preservation while exposed to neutron irradiation.

When exposed to combined simulated space stressors, ambient temperature preservation reduced organoid size, proliferation and increased apoptosis initially, but this returned to unpreserved control levels by 168 h post-exposure resulting in more proliferative cells and less apoptosis (Figure 6 and Figure 7). Moreover, preservation of cardiac organoids while exposed to ^252^Cf radiation and simulated µg seems to enhance beating capacity retainment post exposure (Figure 6).

### 2.5. Enriched GO Biological Process Analysis in Preserved Cardiac Organoids Versus Unpreserved Controls

#### 2.5.1. Ambient Temperature Preservation Promotes a Transcriptional Shift Toward Restoration of Cardiac-Specific Structure and Function

**Figure 8:**
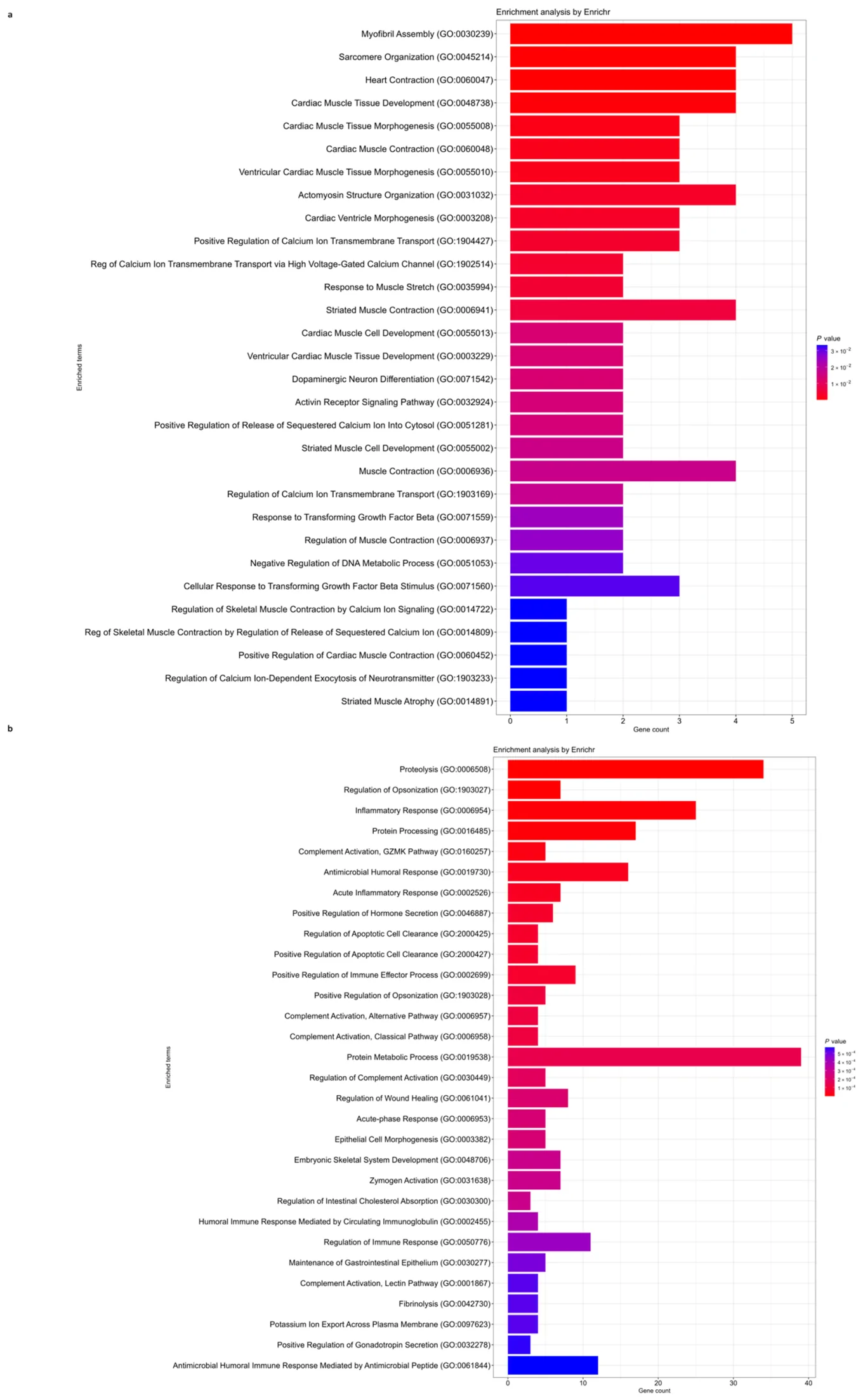
Enrichment analysis of differentially expressed gene sets upon ambient preservation. Enriched biological processes based on (**a**) upregulated and (**b**) downregulated gene sets.

**Figure 9:**
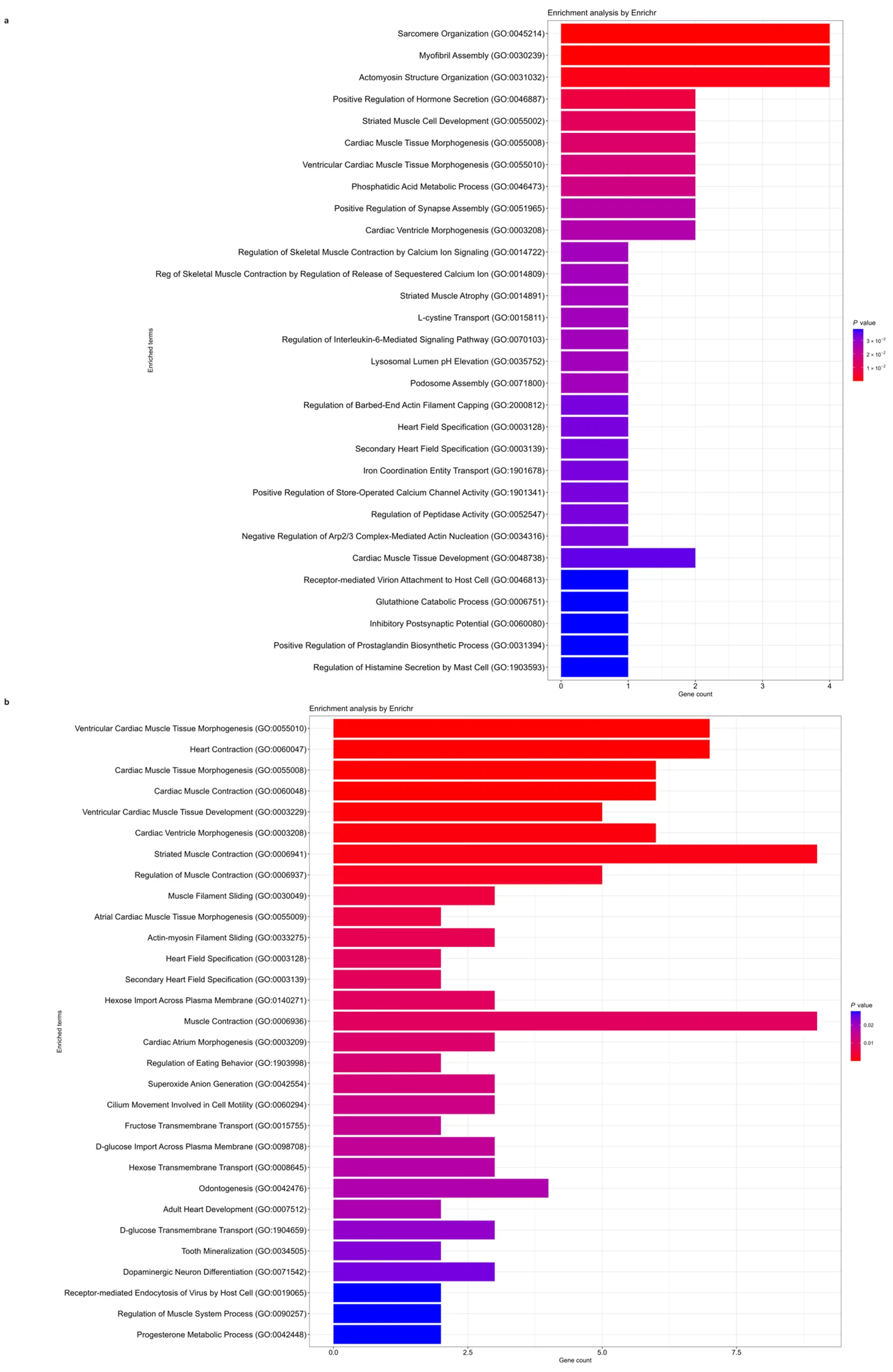
Enrichment analysis of differentially expressed upregulated gene sets upon exposure to (**a**) simulated microgravity (sµg)and (**b**) ^252^Cf radiation, while preserved at ambient temperature.

**Figure 10:**
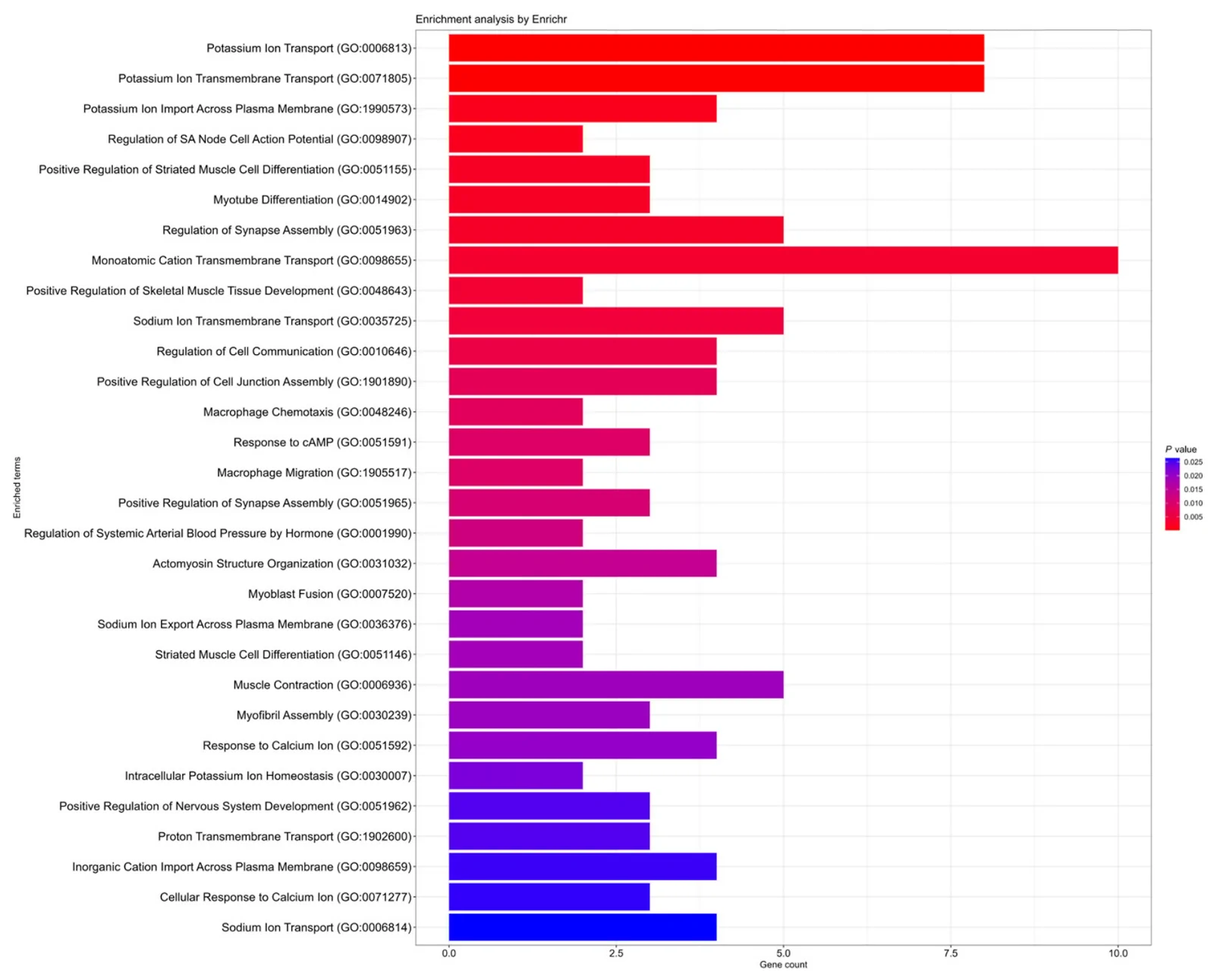
Enrichment analysis of differentially expressed upregulated gene sets upon combined exposure to simulated microgravity (sµg) and ^252^Cf radiation, while preserved at ambient temperature.

Enrichment analysis of the gene sets defined within “GO_Biological_Process_2026” [21] was performed with “Enrichr” [22,23,24] (Figure 8, Figure 9 and Figure 10, Figures S7 and S8). Significant (*p* < 0.05) differentially expressed genes of preserved versus unpreserved conditions, when not exposed to simulated space stressors, were collected in gene sets. Gene ontology (GO) enrichment analysis of RNA sequencing data was performed separately for upregulated and downregulated gene sets following ambient temperature preservation during recovery, revealing distinct and functionally coherent transcriptional responses (Figure 8). Upregulated genes were strongly enriched for biological processes related to cardiomyocyte maturation and structural integrity, including “myofibril assembly”, “sarcomere organization”, and “cardiac muscle contraction”, and driven by key structural and contractile genes including *MYL3*, *MYL4*, *MYL7*, *TNNC1*, *ANKRD1*, and *MYPN* (Figure 8a) [25,26]. In addition, processes linked to calcium ion handling and excitation–contraction coupling (e.g., *CASQ1*, *TRPC3*, *CACNG6*) were enriched, suggesting preservation or reactivation of cardiomyocyte-specific functional pathways (Figure 8a) [27,28,29,30,31]. Developmental and morphogenetic terms, including “cardiac muscle tissue development and morphogenesis”, further indicate engagement of gene programs associated with tissue organization and structural integrity, potentially indicating recovery of the organoid phenotype (Figure 8a) [32,33,34,35]. In contrast, downregulated genes were predominantly associated with immune and inflammatory responses, including complement activation, antimicrobial response, and cytokine signaling, supported by *C3*, *C4A*, *C5*, *CXCL8*, and *TLR2/3* (Figure 8b). This suppression is consistent with prior observations that alteration of innate immune signaling contributes to reduced tissue damage and improved cardiac function following stress or injury [36,37,38,39,40,41]. Furthermore, strong enrichment of proteolysis and extracellular matrix (ECM) remodeling pathways (e.g., *MMP1*, *ADAMTS4*, *PRSS* family members) suggests a suppression of tissue degradation and remodeling processes (Figure 8b). Since excessive ECM turnover and proteolytic activity are hallmarks of pathological cardiac remodeling and fibrosis, their suppression suggests a stabilization of tissue architecture [42,43,44,45,46]. These transcriptional changes are consistent with cardioprotective states observed in ischemia–reperfusion models, where recovery is characterized by restoration of contractile programs alongside suppression of inflammatory and stress pathways [47]. Overall, these findings indicate that recovery upon ambient temperature preservation promotes a transcriptional shift toward restoration of cardiac-specific structure and function, while simultaneously suppressing inflammatory, proteolytic, and stress-associated pathways, consistent with a protective and stabilizing cellular state.

#### 2.5.2. Adaptive Structural Remodeling and Functional Prioritization Under Simulated Microgravity Conditions

Under simulated µg conditions while preserved at ambient temperature, preserved versus unpreserved cardiac organoids when exposed to simulated µg, differential gene expression analysis revealed a clear functional division between upregulated and downregulated biological processes, indicating a shift from systemic metabolic and immune activities toward structural and mechano-adaptive responses. The upregulated gene set was strongly enriched for processes related to muscle structure and contractile apparatus organization, including “sarcomere organization” and “myofibril assembly”, driven by key structural genes including *ANKRD1*, *CASQ1*, *MYPN*, and *LMOD2* (Figure 9a). Additionally, the enrichment of upregulated genes in processes such as “cardiac morphogenesis” and “striated muscle development”, is consistent with established roles of these factors in maintaining contractile integrity and adaptive remodeling of cardiomyocytes under stress conditions (Figure 9a) [14,16,25,26,48,49]. This response aligns with previous studies demonstrating that µg and altered mechanical loading induce cytoskeletal and contractile remodeling as primary adaptive mechanisms in cardiac and muscle cells, including enhanced actin filament turnover and reorganization of actomyosin structures [50,51,52,53,54,55,56,57,58,59,60]. The observed enrichment of pathways regulating actin polymerization, depolymerization, and cytoskeletal organization further supports this interpretation, indicating that cells actively reorganize their structural framework to compensate for reduced mechanical stimuli. Moreover, the upregulation of calcium signaling pathways, particularly those involving intracellular Ca^2+^ handling via *CASQ1*, together with ion transport processes mediated by channels including *ATP1A3* and *KCNH7*, suggests modulation of excitation–contraction coupling and electrophysiological responsiveness, observations that have been widely reported in spaceflight and simulated µg studies of cardiomyocytes [14,19,27,28,29,30,31,61,62]. Collectively, these findings support the notion that µg promotes a structural and mechanoresponsive phenotype aimed at maintaining cellular integrity and functional competence despite altered physical forces [63].

In contrast, the downregulated gene set revealed a marked suppression of metabolic, immune, and extracellular remodeling pathways, highlighting a systemic reduction in energy-intensive and homeostatic processes. Pathways related to lipid metabolism and transport pathways, particularly those involved in “lipoprotein remodeling”, “cholesterol efflux”, and “triglyceride homeostasis” were strongly repressed, with contributions from apolipoprotein genes (*APOA1*, *APOE*, *APOC1-3*, *APOA5*) and lipid regulators including *LPL* and *ANGPTL4* (Supplementary Materials—Figure S7a) [64,65,66]. These observations are consistent with accumulating evidence that µg disrupts lipid metabolism and alters lipoprotein dynamics at both transcriptomic and metabolomic levels. This is supported by studies demonstrating significant alterations in lipid regulatory networks and metabolic homeostasis under spaceflight or simulated µg conditions [65,66,67,68,69,70]. Simultaneously, there was significant suppression of immune and inflammatory responses, including “complement activation” pathways, “acute-phase responses”, and antimicrobial humoral immunity, indicating decreased activity in innate immune defense mechanisms (Figure S7a) [36,37,38,39,40,41,71,72]. Moreover, downregulation of amino acid catabolism and core metabolic pathways further indicates a reduction in metabolic flexibility, a key determinant of cellular adaptability and energy balance in cardiac tissue. In parallel, the marked suppression of immune and inflammatory processes, including complement activation, cytokine signaling, and antimicrobial responses, aligns with well-documented effects of µg and radiation on immune dysregulation and attenuation of innate immune activity. The additional downregulation of proteolysis and ECM remodeling pathways suggests reduced extracellular turnover and tissue remodeling, which may reflect a shift toward structural conservation rather than dynamic reorganization under low mechanical load [16,50,73,74,75].

Overall, these data suggest that under simulated µg while preserved at ambient temperature cardiac organoids prioritize cytoskeletal reorganization, contractile adaptation, and ion-mediated signaling, while simultaneously downregulating energy-intensive systemic functions such as metabolism, immune defense, and extracellular remodeling. This coordinated response likely reflects an adaptive strategy to cope with reduced mechanical load and altered environmental conditions, shifting cellular resources from global physiological processes toward localized structural and mechanosensitive adaptation.

#### 2.5.3. ^252^Cf Radiation Induces Structural Remodeling and Suppresses Tissue Repair Pathways

Functional enrichment analysis of cardioids exposed to ^252^Cf radiation while preserved at ambient temperature compared to unpreserved exposed cardiac organoids revealed a complex transcriptional response characterized by activation of structural repair mechanisms, inflammatory signaling, and metabolic adaptations pathways. The enrichment of upregulated genes in biological processes related to cardiac muscle development, contraction such as “sarcomere organization”, “striated muscle contraction” and “ventricular morphogenesis” (Figure 9b), driven by contractile genes including *MYH6*, *MYBPC3*, *TNNC1*, *MYL3*, *MYL4/7*, is consistent with compensatory responses aimed at restoring contractile integrity following radiation-induced cellular damage. Such activation of cardiomyocyte structural genes has been described in stress and injury models, where enhanced expression of sarcomeric and cytoskeletal elements reflects attempts to maintain or rebuild functional architecture [25,26,76]. In particular, cytoskeletal reorganization and increased actin-myosin filament dynamics are well-recognized responses to mechanical and oxidative stress, supporting cell survival and structural resilience. The enrichment of calcium handling and ion transport pathways, including genes including *CASQ2* and *TPCN2*, further indicates modulation of excitation–contraction coupling and intracellular signaling, processes known to be highly sensitive to oxidative damage (Figure 9b) [77,78,79]. The upregulation of genes involved in ion handling and calcium signaling (e.g., *CASQ2*, *TPCN2*) suggests modulation of intracellular Ca^2+^ dynamics, which are critical for both contractility and stress signaling [1,27,28,29,30,31,80,81,82,83]. Radiation exposure, especially high-LET neutron radiation, induces substantial oxidative stress and perturbs calcium homeostasis, thereby inducing adaptive changes in calcium cycling and electrophysiological regulation [78,83,84,85].

In addition to structural remodeling, the upregulated transcriptional profile included pathways linked to increased energy demand and oxidative stress responses, including monosaccharide transport and ROS-generating systems (*NOX1*, *NOX5*), suggesting a shift toward glycolytic substrate utilization and enhanced redox signaling. This is consistent with radiation biology studies showing that ionizing radiation triggers mitochondrial dysfunction and ROS production, leading to a metabolic shift and increased reliance on readily available energy substrates (Figure 9b) [79,86,87,88,89]. Moreover, the activation of developmental signaling pathways, including *WNT* and *FGF* networks, supports the concept that irradiated cardiac cells may partially re-engage embryonic or regenerative programs, an observation widely reported in cardiac injury models as part of tissue repair and remodeling processes [90,91,92].

In contrast, the biological processes identified within the downregulated gene set, highlight a strong downregulation of ECM remodeling, proteolysis, and inflammatory signaling, indicating that these processes are relatively suppressed in the irradiated condition (Supplementary Materials—Figure S7b). The strongest enrichment is observed in “ECM organization” and “collagen fibril formation”, involving structural genes including *COL1A1*, *COL3A1*, *COL5A3*, *COL12A1*, and remodeling enzymes including *MMP1*, *MMP9*, *MMP13*, *ADAMTS* family members, and *LOXL2/4*. The prominent suppression of ECM-related pathways (including collagens, MMPs, ADAMTS proteases, and integrin-associated signaling) suggests a profound impairment in matrix turnover and remodeling. This is particularly significant because the cardiac ECM provides the structural scaffold required for force transmission and coordinated contraction, and its dynamic remodeling is essential for recovery after injury. Radiation-induced disruption of cell–ECM interactions has been reported to alter mechano-sensing and tissue mechanics, further supporting the interpretation that reduced ECM gene expression reflects impaired mechano-transduction and structural recovery [43,78,93,94,95,96].

In parallel, the downregulation of *IGF1*, *FGF2*, and MAPK-related pathways highlights a suppression of key growth factor networks that regulate cardiomyocyte survival, regeneration, and adaptive hypertrophy. IGF-1 signaling in particular plays a central role in maintaining cardiac homeostasis and resistance to injury, and its dysregulation has been directly linked to radiation sensitivity and adverse cardiovascular outcomes [36,37,38,39,40,41]. Similarly, FGF signaling is known to mediate cardioprotective effects and promote repair after injury, including DNA damage responses and tissue regeneration [97]. Therefore, the coordinated reduction in these pathways strongly suggests diminished regenerative capacity and compromised recovery potential in the irradiated organoids.

The gene set further indicates suppression of anti-apoptotic signaling (e.g., *BCL2*-associated pathways) together with reduced inflammatory and immune responses, including complement activation (*C3*, *C4*, *C5*) and cytokine signaling (*CXCL8*, *CCL2*). While reduced inflammation may transiently limit secondary tissue damage, the concurrent inhibition of apoptotic cell clearance and immune signaling likely impairs removal of damaged cells and disrupts paracrine repair mechanisms. Radiation-induced heart disease is known to involve dynamic phases of inflammation followed by chronic remodeling and dysfunction, and suppression of immune and repair pathways has been associated with impaired healing and increased susceptibility to long-term damage. Thus, the observed transcriptional state is consistent with a late or maladaptive phase of radiation response characterized by reduced regenerative signaling [98].

Altogether, these findings indicate that ^252^Cf exposure while preserved at ambient temperature induces a coordinated response characterized by upregulation of contractile and cytoskeletal remodeling programs, alongside a strong suppression of ECM remodeling, diminished survival and growth signaling, impaired immune-mediated repair, and metabolic adaptation pathways. This transcriptional profile reflects a complex interplay between damage repair, immune activation, oxidative stress handling, and structural reorganization, underscoring the multifaceted cellular response to high-LET radiation.

#### 2.5.4. Combined Simulated Space Stressors Induce Structural Adaptation and Metabolic Suppression

Functional enrichment analysis of cardiac organoids exposed to combined simulated space stressors while preserved at ambient temperature revealed a coordinated and selective transcriptional reprogramming that reflects the integration of mechanical unloading and radiation-induced stress responses (Figure 10).

The upregulated gene set was significantly enriched for biological processes supporting ion transport, membrane excitability, and electrophysiological signaling, including potassium and sodium ion transport mediated by channels encoded by genes including *HCN1*, *HCN4*, *KCNH1*, *KCNH7*, *KCNIP2*, *ATP1A3/4*. These genes are essential for pacemaker activity and regulation of membrane depolarization, and action potential propagation, reflected in enriched terms such as “regulation of sinoatrial node activity”, “cardiac muscle action potentials”, and “membrane depolarization” (Figure 10). Their activation aligns with observations from spaceflight and µg models where cardiomyocytes exhibit altered electrophysiological properties and adaptive modulation of ion channel expression to maintain functional stability [14,16,19,62,99].

Simultaneous enrichment of processes related to cardiac muscle development actomyosin organization and cytoskeletal remodeling, driven by genes including *MEF2C*, *LMOD3*, *MYLK3*, *MYL3*, *MYPN*, suggests that cells actively reinforce structural integrity and contractile capacity in response to combined stressors (Figure 10) [100,101,102]. This is consistent with prior studies showing that both µg and mechanical stress perturbations induce cytoskeletal reorganization and activation of developmental gene programs as compensatory mechanisms to preserve tissue architecture and function [14,60,75,103]. The presence of genes involved in vascular signaling and immune cell recruitment (e.g., *EDN2*, *TAFA4*, and *SELE*) further indicates enhanced intercellular communication and crosstalk between cardiomyocytes and immune or endothelial components, reflecting a stress-responsive signaling network (Figure 10) [63,75,102,104,105,106]. Altogether, these upregulated pathways imply an adaptive shift toward enhanced electrophysiological responsiveness, cytoskeletal remodeling, and intercellular communication, potentially indicating compensatory mechanisms to maintain tissue function under altered gravitational and radiation stress.

In contrast, the downregulated gene set revealed a suppression of lipid metabolism, systemic metabolic activity and immune-related processes, indicating a broad suppression of energy-intensive and homeostatic functions. A strong suppression of lipid metabolism and transport processes was observed, including “triglyceride and cholesterol homeostasis” mediated by apolipoproteins (*APOA1*, *APOE*, *APOC1-3*, *LPL*, *ANGPTL3/4*, *DGAT2*, *MLXIPL*), as well as “fatty acid and sterol metabolic pathways” (Supplementary Materials—Figure S8) [64,65,66,107,108]. This is in line with accumulating evidence that radiation and simulated µg disrupt lipid handling and metabolic homeostasis at both transcriptomic and functional levels. Microgravity exposure has been shown to alter lipid regulatory networks and reduce metabolic efficiency, contributing to impaired energy storage and substrate utilization [19,65,66,67,68,69,70]. The simultaneous downregulation of amino acid catabolism and xenobiotic metabolism pathways (e.g., *CYP*, *ADH*, *ARG1*, *PAH*) further supports the concept of reduced metabolic flexibility, a hallmark of cellular stress and dysfunction in cardiac systems. Such suppression suggests an impaired ability to switch between energy substrates, consistent with established links between disrupted amino acid metabolism and compromised cardiac energetics [107,109,110,111].

Additionally, the observed reduction in complement activation, cytokine signaling, and innate immune pathways indicates altered immune responsiveness under combined sµg and radiation exposure. While radiation alone is typically associated with immune activation and inflammation, the attenuated immune signature observed here may reflect a complex interplay between µg-induced immune suppression and radiation-driven immune signaling, resulting in a rebalanced or dysregulated immune state as reported in space biology research. The concurrent downregulation of proteolytic pathways and ECM turnover further suggests reduced tissue remodeling and circulatory system activity, potentially indicative of suppressed systemic-like functions in the organoids under these combined stress conditions [41,71,72,94,112,113,114,115].

Overall, the transcriptional profile under these combined stressors is characterized by a division between upregulated electrophysiological, developmental, and signaling processes and downregulated metabolic, hemostatic, and immune pathways, highlighting a coordinated reprogramming that prioritizes cellular communication and structural adaptation, while affecting systemic metabolic and circulatory homeostasis. However, RT-qPCR validation of the described results was not included in the study design, which is a limitation of the current study. While bulk RNA sequencing provides a comprehensive overview of transcriptional changes, validation of selected differentially expressed genes using an independent method would strengthen confidence in the reported findings. Therefore, these interpretations should be regarded as hypothesis-generating and validated in future targeted functional studies.

## 3. Discussion

**Figure 11:**
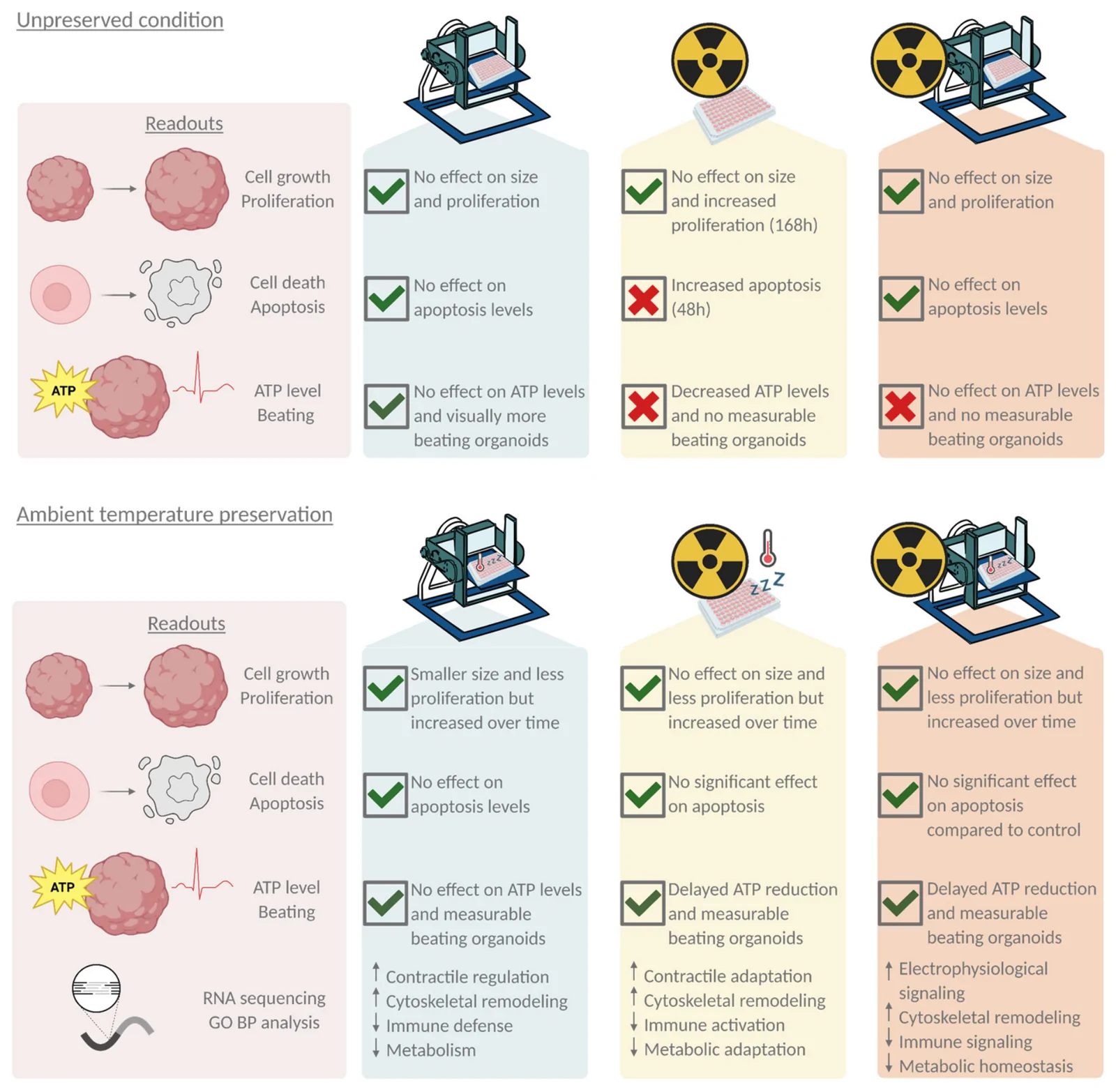
Graphical overview representing the main findings based on our study design. The conclusions drawn from the present findings should be interpreted in light of the limitations of the study design. Arrows represent up- and down-regulated processes. Green check symbol refers to a beneficial effect. Red cross symbol refers to an adverse effect. Created in BioRender. Bijnens, M. (2026) https://BioRender.com/vdvgft3.

Preserved and unpreserved cardiac organoids were exposed to RPM-simulated µg and/or an accelerated mixed neutron–photon field generated by ^252^Cf to assess viability and function (Figure 11). RPM exposure alone produced no statistically significant changes in selected readouts under the conditions tested. In contrast, ^252^Cf exposure altered ATP levels, proliferation, apoptosis and contractile readouts, with some changes appearing earlier in unpreserved organoids. Preserved irradiated organoids retained measurable beating during the recovery period, whereas beating could not be quantified in unpreserved irradiated samples. Preservation itself nevertheless caused transient changes in size, proliferation, and beating parameters. The findings therefore suggest that the ambient-preservation condition supports post-exposure recovery of contractile activity, but they do not by themselves demonstrate a direct radioprotective mechanism. Combined exposure was associated with changes in size, ATP levels, and contractile timing, formal interaction testing will therefore be required to determine whether radiation and simulated µg act additively, synergistically, or antagonistically. The distinct temporal proliferation patterns observed between preserved and unpreserved organoids may reflect differences in the recovery dynamics following preservation and subsequent exposure. Unpreserved organoids exhibited higher proliferative activity during the early recovery phase, whereas preserved organoids showed a more gradual increase in proliferation over time. One possible explanation is that preservation induces a transient reduction in proliferative activity, delaying cell-cycle re-entry while cells prioritize restoration of cellular homeostasis and repair of preservation- and exposure-induced stress. Such a delayed proliferative response may be particularly relevant following additional stressors such as simulated microgravity or radiation, which can activate cellular stress and DNA damage response pathways. Consequently, preserved organoids may require a longer recovery period before resuming active proliferation, resulting in lower proliferation levels at earlier time points but progressive recovery at later stages. Although this interpretation is consistent with trends observed in our previous study using the CellShip^®^ preservation platform [20,116], the underlying molecular mechanisms were not investigated in the present work and therefore remain speculative. Further studies examining cell-cycle regulation, stress-response pathways, and DNA repair processes are needed to elucidate the biological basis of these temporal differences in proliferative behavior.

Previous studies investigating the effects of simulated and µg on cardiac cells have reported variable outcomes that depend strongly on the model system and exposure conditions. Experiments performed under µg-like conditions, such as those conducted onboard the ISS, have demonstrated enhanced proliferation and structural growth in 3D hiPSC-derived cardiac constructs, while largely preserving functional properties such as beating activity [14,60]. In contrast, simulated µg platforms such as a 2D clinostat or the RPM have yielded more heterogeneous results, including altered calcium handling and contractility, oxidative stress, or reduced proliferation, but often without pronounced effects on overall viability [60,61,117,118]. Even enhanced tissue maturation features were observed in 3D hiPSC-cardiomyocytes upon seven and fourteen days on the RPM [48]. These observations are consistent with our findings, where RPM exposure alone did not induce significant changes across the evaluated readouts, suggesting that 75 h exposure to simulated µg may not be sufficient to compromise viability or function in this cardiac organoid model. However, our findings are specific to the cardiac organoid model, simulated microgravity platform, and exposure duration employed, which may limit their generalizability to other experimental systems. The effects of (simulated) microgravity on cardiac function are known to vary depending on factors such as tissue complexity, cellular composition, exposure conditions, and the functional endpoints assessed. Consequently, comparisons with previous studies should be interpreted with caution, as methodological differences may contribute to divergent observations.

In contrast, the biological effects of ionizing radiation, particularly high-LET neutron radiation, are consistently associated with cytotoxic outcomes. Studies using ^252^Cf radiation on cultured human cells have demonstrated clear dose-dependent reductions in cell survival and increased mutation rates, with a higher relative biological effectiveness (RBE) compared to photon irradiation, including γ-ray radiation [119,120]. Mechanistically, neutron radiation produces dense ionization tracks and complex DNA damage that are difficult to repair, ultimately leading to increased apoptosis and loss of proliferative capacity. While studies combining neutron irradiation with cardiac 3D models remain scarce, complementary evidence from space-relevant radiation types, including protons and photons, supports similar conclusions. In this context, radiation exposure has been shown to induce oxidative stress [76], cardiomyocyte apoptosis and alter cardiac function, with ATP-dependent mechanisms playing a protective role through modulation of autophagy [121]. More recent work further demonstrates that irradiation directly triggers apoptotic signaling and structural damage in cardiac cells, which can be mitigated by ECM components, highlighting apoptosis as a central response pathway [122]. In addition, radiation-induced cardiomyocyte damage has been linked to mitochondrial dysfunction, inflammasome activation, and dose-dependent reductions in cell viability, reinforcing the sensitivity of cardiac cells to ionizing radiation [15,123,124,125]. Functional alterations have also been observed at the beating level, where X-ray irradiation modifies conduction properties and mitochondrial density in cardiomyocytes [126,127].

Taken together, the current literature indicates that while simulated µg alone may exert relatively modest effects on cardiac cell viability, ionizing radiation, particularly high-LET radiation such as neutron exposure, represents a dominant driver of cellular damage. This is consistent with our observations, where little significant effects of simulated µg exposure were detected, whereas clear radiation-induced alterations were observed across the included viability- and function-related readouts. Furthermore, the limited availability of studies investigating neutron radiation, such as ^252^Cf, in complex 3D cardiac systems highlights an important gap in the field. Our findings therefore contribute as a proof-of-concept to bridging this gap by demonstrating radiation sensitivity in a physiologically relevant cardiac organoid model and highlight the need for further studies integrating advanced 3D systems with space-relevant radiation and µg analogs to evaluate individual and potential synergistic effects.

In addition to the evaluation of the effects of simulated space stressors, both individually and combined, the effects of the ambient temperature preservation itself were considered. Ambient temperature preservation alone caused a temporary reduction in proliferation and a minor decrease in organoid size, particularly under simulated µg, but the cardioids recovered over time. Similarly, preservation also delayed proliferative capacity following high-LET radiation exposure, but this was restored to control levels at later timepoints. Despite these initial effects, preserved irradiated organoids retained beating capacity, whereas beating could not be measured in unpreserved irradiated organoids, suggesting a protective effect of preservation during radiation exposure. A similar benefit was observed under combined radiation and simulated µg, where preserved organoids maintained beating function despite transient reductions in size and proliferation and increased apoptosis.

GO biological process enrichment analyses generated hypotheses consistent with the phenotypic findings. Preservation was associated with enrichment of gene sets related to cardiac structure and function and with reduced representation of inflammatory processes. Simulated µg may be associated with cytoskeletal, contractile, and ion signaling gene sets, and ^252^Cf radiation may be associated with stress-related, ECM, survival and immune-response gene sets. Combined exposure was associated with transcriptional changes involving electrophysiological, structural, metabolic, and immune-related pathways, although these observations require further validation. Because over-representation analysis identifies associations within a selected differentially expressed gene list and does not establish pathway activation, cellular adaptation, or causal protection, these interpretations should be regarded as hypothesis-generating and validated in future targeted functional studies. Although RNA sequencing provides a comprehensive overview of gene expression changes, additional validation, including RT-qPCR, would strengthen confidence in the identified differentially expressed genes. Furthermore, the transcriptomic analyses were specifically designed to compare preserved and unpreserved organoids within each exposure condition and were not intended to comprehensively characterize the biological effects of simulated microgravity or radiation. Consistent with this focused study design, no significantly enriched pathways directly related to proliferation, ATP metabolism, or apoptosis were identified that could readily explain the observed phenotypic outcomes. Consequently, the relationship between the transcriptomic and functional findings remains associative rather than mechanistic. Future studies incorporating RT-qPCR validation, targeted analyses of radiation- and microgravity-induced responses, and complementary metabolic and functional assessments will be important to further elucidate the molecular basis of the observed effects.

Previous studies have demonstrated that 3D organoid systems and stem cell-derived tissues can be successfully preserved and transported under static conditions, including during flight-like or shipping scenarios [19]. In this context, human stem-cell derived organoids maintained high viability (±95%), stable apoptosis levels, and preserved electrophysiological activity following multi-day transport in sealed, medium-filled containers [128,129]. In cardiac models, preservation strategies have further shown that functional readouts such as beating activity can recover after storage, even when temporarily halted during preservation, indicating that transient functional arrest does not necessarily reflect irreversible damage [130,131]. These findings highlight the importance of environmental control, including container filling, temperature stability, and minimization of shear stress, for maintaining viability and function in complex 3D systems. In the context of space research, preservation is a critical requirement for enabling transport and delayed experimental execution onboard the ISS. Cardiac spaceflight experiments consistently rely on pre-preservation strategies, most commonly cryopreservation. For instance, cryopreserved 3D hiPSC-derived cardiac progenitor spheroids have been successfully transported to the ISS, thawed in orbit, and cultured for extended periods, resulting in viable, proliferative, and functionally active cardiomyocytes exhibiting spontaneous beating [14]. The underlying methodology, including combining cryopreservation with CO_2_-independent culture systems, was specifically developed to address logistical constraints of spaceflight, such as limited crew time, absence of standard incubators, and the need for stable, closed systems [132]. More recent studies using hiPSC-derived cardiac spheroids further confirm that 3D cardiac constructs can be transported to the ISS and retain beating activity and metabolic responsiveness following exposure to µg [133].

Altogether, these studies demonstrate that preserved 3D cardiac tissues can withstand transport, storage, and delayed activation while maintaining viability, proliferative capacity, and functional properties. However, current approaches almost exclusively rely on cryogenic storage, using toxic CPAs and requiring specialized infrastructure. In contrast, ambient temperature preservation strategies remain largely unexplored in the context of spaceflight. In this regard, our findings provide a proof-of-concept for a complementary or alternative approach by demonstrating that 3D cardiac organoids can be maintained under ambient conditions, while preserving viability and functional properties after recovery. This suggests potential advantages for simplifying logistics, reducing reliance on cryogenic infrastructure, and expanding experimental flexibility in future space biology applications. In particular, understanding the impact of space-related stressors on the cardiovascular system is essential for assessing astronaut health risks during long-duration missions [14,49,52,134,135]. However, maintaining the viability and functionality of complex cell cultures during transport to the ISS or during deep-space missions remains a major logistical and technical challenge, often requiring substantial resources for preservation, storage, and handling. The development of ambient temperature preservation strategies that maintain both viability and function of 3D cellular systems could significantly reduce current technical barriers and enable more flexible experimental designs in space biology. In our study design, ambient temperature preservation was applied on 3D cardiac organoids for seven days while exposed to simulated space stressors by using the RPM and high-LET ^252^Cf radiation. This timeframe is particularly relevant, as it aligns with typical payload preparation and transport durations for space missions, including those to the ISS [136,137]. Consequently, room temperature-based preservation may offer practical advantages by simplifying handling procedures, reducing dependence on cryogenic infrastructure, and lowering payload weight and associated costs. Therefore, future studies should focus on scaling these preservation approaches to larger and more integrated tissue systems, as well as evaluating their long-term stability under combined space-relevant stressors, including prolonged exposure to µg and an extended spectrum of radiation simulating cosmic radiation.

While room-temperature preservation using CellShip^®^ has previously been validated for suspended cells for up to 120 h in static conditions, we recently published a study that extends this approach to a more complex 3D cell model over seven days [20]. Our findings suggested potential applicability of this method over longer durations and in more advanced biological systems, although this will require further validation through adapted experimental designs. In addition, the influence of scaling and container-related parameters should be considered in the context of ambient temperature preservation and transport. Factors such as container geometry, volume-to-tissue ratio, and gas exchange may affect preservation outcomes. In the current study, cardiac organoids were preserved in air-tightly sealed 96-well plates, which were placed at the side or on the RPM to simulate µg. This experimental set-up was selected to maintain stable conditions, prevent medium evaporation, and enable a simple, low-effort workflow requiring minimal handling and small volumes of preservation medium. The use of 96-well plates also aligns with the standard culture format of the organoids, as they are generated, matured, and maintained in the same system, thereby reducing potential sources of variability. However, this simplified setup does not account for potential effects of medium exchange during storage and does not systematically address variables relevant to scaling, such as container type, volume, or oxygen availability. Modifications to these parameters, for example, periodic medium replacement or the use of alternative container formats, may influence long-term preservation outcomes. A systematic evaluation of these factors was beyond the scope of our study. Furthermore, a limitation of the present study is the absence of a conventional cryopreservation control group. Although inclusion of cryopreserved organoids would have enabled direct comparison with the current standard preservation approach, incorporating this additional condition was beyond the scope of the study due to the substantial increase in experimental complexity, sample requirements, and logistical demands associated with the tightly synchronized exposure procedures. In addition, cryopreservation was intentionally not included because previous work from our group demonstrated that this preservation approach resulted in suboptimal recovery of cardiac organoids following thawing, with adverse effects on both viability and functional performance [20]. As the aim of the present study was to evaluate the feasibility of an ambient-temperature preservation strategy as an alternative to conventional cold-chain dependent approaches, comparisons were therefore restricted to room-temperature-preserved organoids and fresh, unpreserved controls. Consequently, the current findings cannot provide insight into how cryopreserved organoids would respond to simulated spaceflight-associated stressors, nor whether cryopreservation itself would modify susceptibility to radiation exposure or simulated microgravity. Cryopreservation may introduce additional cellular stress, alter metabolic activity, or affect tissue integrity, all of which could potentially influence subsequent responses to environmental challenges. Future investigations should therefore include a cryopreserved control group and directly compare fresh, ambient-temperature-preserved, and cryopreserved organoids under identical spaceflight-relevant conditions. Such studies would enable a more comprehensive assessment of the relative advantages and limitations of each preservation strategy, while also determining whether preservation-induced alterations influence biological outcomes following exposure to simulated space stressors.

Another limitation of this study is its exclusive focus on cardiac organoids derived from a single hiPSC line, as our model of interest, which restricts the generalizability of the findings. Therefore, these results should be considered as being preliminary. Further investigations are needed to evaluate whether the observed effects can be replicated across multiple hiPSC lines and extended to a wider variety of microtissue models. Furthermore, organoid size and morphology were not quantitatively assessed immediately prior to the simulated space stressor exposure. Although the cardiac organoids were generated using a standardized and validated protocol designed to produce highly uniform constructs [13,20], and organoids were randomly assigned to experimental groups following routine quality control assessments, the absence of direct size measurements before exposure precludes complete exclusion of size-related variability between groups. Future studies should incorporate systematic imaging and quantitative morphometric analyses before and after preservation and exposure to further strengthen the evaluation of treatment-related effects on organoid structure and function.

In this study, cell viability was primarily assessed through ATP quantification using the CellTiter-Glo^®^ 3D Cell Viability Assay and functional beating capacity. Supporting indicators included the presence of proliferative (Ki67-positive) cells and the proportion of apoptotic (CC3-positive) cells, which together provided insight into viability within the cardiac organoid model. However, incorporating additional methods, including direct cell counting and fluorescence-based live/dead imaging, would strengthen the evaluation of cell viability, cell loss, and cell death. While the included assays provide robust measures of overall viability and function, they do not offer spatial information on the distribution of viable and non-viable cells within the 3D organoid structure. Additional approaches such as FDA/PI staining combined with advanced 3D imaging could provide a more comprehensive assessment of cellular viability. However, these methods require specialized live-imaging infrastructure and rapid post-exposure analysis, which were beyond the scope of the current study. Future investigations incorporating such techniques would further strengthen the characterization of organoid responses to preservation and spaceflight-relevant exposures. Furthermore, the observed changes in individual beating parameters cannot directly be classified as beneficial or detrimental for this model. The MUSCLEMOTION software tool offers a quantitative assessment of organoid movement as a mechanical response, which allows the detection of beating behavior alterations without assessing functional superiority based on specific parameter shifts when evaluating the output. Our interpretation is based on the overall deviation from control behavior. Therefore, assigning functional superiority to specific parameter values is not possible. Furthermore, organoid beating dynamics were quantified using this tool without complementary electrophysiological, calcium-handling, or contractility measurements. Although this approach enables objective assessment of changes in contraction behavior, alterations in parameters such as contraction duration, relaxation time, or beating frequency cannot be directly interpreted as indicators of improved or impaired cardiac function. Additionally, temporal changes observed during the recovery period may reflect adaptive responses to exposure rather than preservation-specific effects. Consequently, the physiological significance of the observed beating alterations remains uncertain. Future studies incorporating more comprehensive functional analyses will be necessary to clarify the biological relevance of these findings and to better understand the effects of preservation and simulated microgravity on cardiac organoid performance.

Although our findings indicate a potential for ambient temperature preservation of 3D cell constructs for space-related biological experiments, additional validation is required before drawing broader conclusions about its overall applicability. Our study design aimed to simulate key space-related stressors, including µg and radiation, that are relevant to astronaut exposure. Microgravity-like conditions were simulated using an RPM, which induces a continuous free-fall state. While this approach mimics certain aspects of µg, it does not fully replicate the complexity of true µg environments. The RPM is a 3D free-fall simulator that rotates along two axes (x-axis and y-axis), each at different and random speeds [7]. In this way, the apparent resulting gravitational vector is reduced to zero. Simulation of this µg condition requires that the rotation of the independent frames of the RPM must be faster than the biological processes occurring in the cells, while limiting centrifugal forces [6]. Moreover, the use of the RPM introduces technical challenges in cellular experiments, including limited sample capacity and the need to minimize shear stress caused by air bubbles. However, the mechanical environment generated by the RPM is complex and consists not only of gravity-vector averaging, but also fluid motion, shear stress, and rotational accelerations. The relative contribution of these factors strongly depends on the culture configuration. To address these issues, no gel or matrix coating was applied to the culture plates, as prior in-house experience indicated that these approaches could introduce technical complications during sample collection and lead to sample loss. Instead, 96-well plates were carefully filled and sealed to minimize the presence of air bubbles and likely limit organoid displacement, fluid circulation, and shear stress during RPM operation [6,138]. Under these conditions, the RPM may primarily induce continuous reorientation of the gravity vector while generating little biologically relevant unloading. This may be particularly important for cardiac organoids, as the contractile forces produced by beating cardiomyocytes are expected to be orders of magnitude greater than gravitational loading acting on the tissue [60]. Although RPM studies have been conducted using adherent cells, free-floating spheroids, and organoids, these systems are generally investigated separately [139]. To date, no systematic study has directly compared identical biological models cultured as free-floating, matrix-embedded, and substrate-attached preparations under identical RPM conditions. Therefore, it remains unclear to what extent reported biological responses result from simulated µg itself or from differences in mechanical coupling between the sample, ECM, and surrounding fluid.

Although RPM systems are widely employed to reproduce aspects of gravitational unloading by continuously randomizing the gravity vector, they do not fully recapitulate the fluid dynamic environment encountered during spaceflight. In true microgravity, the absence of buoyancy-driven convection results in a diffusion-dominated environment in which nutrient delivery, waste removal, and gas exchange occur primarily through passive transport mechanisms [140,141,142]. In contrast, the continuous multi-axis rotation of the RPM induces fluid movement and mixing within the culture medium, potentially enhancing mass transport and reducing the formation of diffusion gradients around the organoids. Consequently, cellular oxygenation and nutrient availability may differ substantially between RPM-exposed samples and both static 1g controls and authentic spaceflight conditions [138]. These differences are particularly relevant when interpreting the combined RPM and irradiation experiments. Oxygen availability is a well-established determinant of radiation sensitivity, as molecular oxygen can enhance the fixation of radiation-induced DNA damage through the oxygen enhancement effect [143,144]. Therefore, RPM-induced alterations in oxygen transport may have influenced the magnitude of the observed radiation responses, including changes in ATP levels, proliferation, apoptosis, and gene expression. As oxygen tension was not directly measured in the current study, it is not possible to determine the extent to which the observed effects reflect responses to gravitational unloading itself, altered mass transport conditions, or interactions between oxygen availability and irradiation. The absence of significant effects on viability and beating capacity following RPM exposure alone may similarly reflect, at least in part, the unique fluid dynamic environment generated by the RPM. These considerations highlight the need for caution when extrapolating RPM findings to true spaceflight conditions. More broadly, the interplay between oxygenation, altered gravity, and radiation exposure represents an important challenge for future human space exploration. Understanding how local oxygen availability influences cellular responses to radiation under conditions of altered gravity will therefore be critical for predicting tissue adaptation and radiation risk during long-duration spaceflight. Future studies incorporating direct measurements of oxygen tension, mass transport dynamics, and validation in real microgravity platforms will be essential to disentangle these interconnected factors and improve the translational relevance of ground-based spaceflight analog models. Collectively, these considerations suggest that the limited effects observed in our cardiac organoids may reflect both the intrinsic biomechanics of the model and the constraints of RPM-based µg simulation in small-volume culture systems. Future validation in low Earth orbit (LEO) would be ideal to distinguish between intended effects and artifacts induced by the model’s limitations, and thus to further validate the quality of RPM simulations.

Furthermore, a ^252^Cf source was used to simulate the neutron component of GCR. Although astronauts are primarily exposed to high-energy protons, heavy ions, and secondary neutrons, the latter are of particular concern for deep-space missions due to their high penetrative capacity and contribution to secondary radiation. Radiation from a ^252^Cf source is widely accepted as a space radiation analog, as it produces highly damaging radiation per unit energy and is more difficult to shield against. The neutron irradiation conditions used in this study resulted in a cumulative dose equivalent of approximately 92.5–99.5 mSv delivered at a dose rate of approximately 1.3 mSv/h. Although this dose rate is considerably higher than that encountered during human spaceflight, the total accumulated dose is within a range relevant to long-duration exploration missions. Astronauts aboard the ISS typically receive approximately 80–160 mSv during a six-month mission, corresponding to average dose rates of about 0.3–1 mSv/day [145]. In contrast, missions beyond LEO are expected to result in substantially higher cumulative exposures due to the absence of geomagnetic shielding [86,146]. Measurements performed by the Radiation Assessment Detector aboard the Mars Science Laboratory during transit to Mars reported an average radiation dose equivalent of approximately 1.8 mSv/day in deep-space [147], which would result in cumulative exposures approaching 1 Sv during a round-trip Mars mission [146,148]. Compared with these values, the cumulative neutron dose administered in our study is comparable to that received during a typical six-month ISS mission, but delivered over a much shorter period. Consequently, our experimental design does not accurately replicate the chronic low-dose-rate radiation environment experienced during spaceflight, but instead represents an accelerated exposure model that enables the investigation of radiation-induced biological responses and the potential radioprotective effects of ambient preservation in cardiac organoids within experimentally practical timescales. Such accelerated irradiation strategies are commonly applied in ground-based space radiobiology studies to model cumulative radiation effects while maintaining feasible experimental durations [86,146]. Future studies will be crucial to determine whether this approach can reliably complement existing preservation strategies used to send cellular experiments to the ISS or beyond and provide practical benefits in specific experimental study designs for future space experiments.

To compare the responses to different radiation qualities, acute ^60^Co γ-ray irradiation was evaluated in addition to accelerated ^252^Cf exposure (Supplementary Materials). ^60^Co γ-rays are low-LET radiation, whereas the ^252^Cf source produces a mixed field in which neutrons generate densely ionizing recoil particles and generally show higher RBE than photons [149,150]. The ^60^Co and ^252^Cf experiments differed not only in radiation quality but also substantially in absorbed dose, dose rate, and exposure duration; direct attribution of between-experiment differences to LET or chronicity alone is therefore not possible. Irradiation with a ^60^Co source resulted in comparatively limited effects on cardiac organoid viability and functional properties, whereas exposure to ^252^Cf induced more pronounced alterations across multiple readouts. This difference is consistent with the fundamental radiobiological properties of these radiation types. ^60^Co emits low-LET γ-rays, which primarily induce sparsely distributed DNA damage that is often repairable; whereas, ^252^Cf emits high-LET neutrons that generate dense ionization tracks, leading to complex DNA lesions that are more difficult to repair and thus exhibit higher RBE [149,150]. In addition, ^252^Cf generates a mixed radiation field with both neutron and gamma components, more closely resembling the heterogeneous radiation environment encountered in space [86,151]. As mentioned before, neutron radiation is a relevant component of the space radiation environment, particularly as secondary radiation generated by interactions of GCRs with shielding materials, further supporting the use of ^252^Cf as a ground-based analog [152]. In addition to differences in radiation quality, the exposure profile likely contributed to the observed effects. In this study, ^60^Co irradiation was delivered as an acute dose, whereas ^252^Cf exposure occurred chronically at low dose rates. Chronic irradiation has been shown to result in cumulative biological damage through sustained oxidative stress, persistent DNA damage signaling, and impaired cellular recovery mechanisms, even at relatively low dose rates [8,153]. Moreover, prolonged low-dose neutron exposure may better reflect the continuous radiation environment encountered during long-duration space missions, in contrast to acute γ-irradiation exposure that do not capture this temporal aspect. Together, these findings are compatible with established differences in radiation quality, but the ^252^Cf field represents only one accelerated ground-based model of a secondary-neutron component and does not reproduce the full mixed particle spectrum or low dose rates encountered in space. Future studies using matched absorbed doses, dose rates, and exposure schedules, together with reporting of neutron and photon dose components, will be needed for a rigorous comparison of radiation quality.

Overall, this proof-of-concept study provides preliminary evidence that ambient-temperature preservation in a microplate format can maintain recovery of cardiac organoid viability and contractile activity following exposure to selected simulated space stressors. However, given the exploratory nature of the study and its limitations, additional validation is required before broader conclusions can be drawn. Future studies should assess robustness across storage durations and temperature excursions, distinguish the contributions of medium composition, temperature, and metabolic suppression, and evaluate the reproducibility and generalizability of these findings across a wider range of preservation conditions and spaceflight-relevant environments. 

## 4. Materials and Methods

### 4.1. Organoid Culture

Cardiac organoids were generated from human-induced pluripotent stem cells (hiPSCs) (UCSD163i-95-1, WiCell Research Institute, Madison, WI, USA) and maintained at 37 °C in a humidified atmosphere containing 95% air and 5% CO_2_. Cardiomyocyte differentiation was performed on the basis of the differentiation protocol previously published by Hofbauer et al. with adaptations [12,13]. Cardioids are first formed through an eight-day differentiation protocol including mesoderm induction, cardiac mesoderm induction and cardiomyocyte specification. Cardioids are then dissociated and reassociated into a Corning^®^ 96-well black/clear round bottom ultralow attachment surface spheroid microplate (Corning, 4520, Berlin, Germany) to form the final organoids. Cardiac organoids were maintained in maintenance medium, chemically defined medium (CDM) prepared by mixing 50% IMDM (GIBCO^TM^, 12440053, Thermo-Fischer Scientific, Waltham, MA, USA) and 50% F-12 Nutrient Mix (GIBCO^TM^, 11765054, Thermo-Fischer Scientific, Waltham, MA, USA) and adding 5 mg/mL BSA (Sigma-Aldrich, A7030, Merck KGaA, Darmstadt, Germany), 1% chemically defined concentrated lipids (GIBCO^TM^, 11905031, Thermo-Fischer Scientific, Waltham, MA, USA), 0.004% 1-thiolglycerol (Sigma-Aldrich, M6145, Merck KGaA, Darmstadt, Germany) and 15 µg/mL Transferrin (Sigma-Aldrich, 178481, Merck KGaA, Darmstadt, Germany) with subsequent 0.2 µm filter filtration. This CDM was supplemented with 10 µg/mL insulin (Roche, 11376497001, Merck KGaA, Darmstadt, Germany) and 100 ng/mL VEGF-165 (STEMCELL, 78159.1, Saint Égrève, France). Size measurements based on the cardioid diameter were performed via brightfield images taken with the Leica Application Suite microscope software platform V4.13 (Leica Microsystems, Wetzlar, Germany).

### 4.2. Preservation

Ten-day-old cardiac organoids were transferred to a Corning^®^ 96-well Black/Clear Round Bottom Ultra-Low Attachment Surface Spheroid Microplate (Corning, 4520, Berlin, Germany), containing 300 µL of CellShip^®^ Cell Transport medium without insulin (Life Science Production, SCS-004K, Bedfordshire, UK) and a single cardiac organoid per well. These plates were sealed using sterile Nunc^™^ Seals (Thermo-Fisher Scientific, 236366, Waltham, MA, USA) to prevent medium evaporation and stored in a transport box to prevent vibrations for preservation at room-temperature (20–25 °C). After exposure to simulated space stressors, medium was changed with 200 µL prewarmed maintenance medium per well, with subsequent medium changed every 48 h. Two days (48 h), four days (96 h) and one week (168 h) post-exposure, viability and functionality measurements were performed. Unpreserved controls were transferred to the same type of 96-well plates, with 300 µL maintenance medium, maintained under standard culture conditions in a humidified incubator and directly subjected to the subsequent experimental exposures. Prior to exposure they were sterile sealed and kept at 37 °C while exposed to simulated space stressors.

### 4.3. Space Stressor Simulation

#### 4.3.1. Simulated Microgravity

Simulated µg (sµg) conditions were created using an RPM 2.0 (Yuri Gravity GmbH, Meckenbeuren, Germany). The well plates were filled with maintenance or preservation medium and sealed with a sterile sealer, allowing airtight and bubble-free sealing of the 96-well plates, as air bubbles are a cause of concern regarding µg experiments due to the impact of shear stress on the cells. The cardioids were exposed to simulated µg for 75 h to evaluate cellular responses to chronic sµg exposure. Although RPM systems are widely employed to reproduce aspects of gravitational unloading by continuously randomizing the gravity vector, they do not fully recapitulate the fluid dynamic environment encountered during spaceflight.

#### 4.3.2. Ionizing Radiation Exposure

A Californium-252 (^252^Cf) bare mixed neutron–photon source (Hopewell Designs, Alpharetta, GA, USA) was used to chronically irradiate preserved and unpreserved cardiac organoids for 70.5–74.7 h, depending on experimental run, in free air with a panoramic beam at a distance of 75 cm. Dosimetry was performed in accordance with ISO 8529-1:2021 (Neutron reference radiations fields. Part 1: Characteristics and methods of production), ISO 8529-2:2000 (Reference neutron radiations. Part 2: Calibration fundamentals of radiation protection devices related to the basic quantities characterizing the radiation field) and ISO 8529-3:2023 (Neutron reference radiation fields. Part 3: Calibration of area and personal dosemeters and determination of their response as a function of neutron energy and angle of incidence). The samples were located in the center of a 10 m × 10 m × 5 m low scattering room (Laboratory of Nuclear Calibration (LNK) at Belgian Nuclear Research Centre (SCK CEN), Mol, Belgium). The RPM system was placed in a standard dry incubator at 37 °C or at ambient temperature, depending on the condition. These incubators were situated at the height of the radiation outlet using a scaffold. An average dose equivalent rate of 1.31–1.33 mSv/h was applied, resulting in a final cumulative dose of 92.5–99.5 mSv over 70.5–74.7 h, respectively.

In addition, unpreserved and preserved cardiac organoids were irradiated vertically in free air by gamma-rays (γ-rays) using a Cobalt-60 (^60^Co) γ-ray source (1.1732, 1.3325 MeV) with a 35 cm × 35 cm collimated beam at a distance of 100 cm. Dosimetry was performed in accordance with ISO 4037:2019 (Radiological protection—X and gamma reference radiation for calibrating dosemeters and doserate meters and for determining their response as a function of photon energy. Part 1: Radiation characteristics and production methods). Cardiac organoids were irradiated with a total dose of 1 Gy using a dose rate of approximately 0.62 Gy/min. Results of radiation experiments using ^60^Co are described in the Supplementary Materials (Figures S3–S6) and referred to in the Section 3.

### 4.4. Immunofluorescence Imaging

#### 4.4.1. Cryosections

Cardiac models were fixed with 10% neutral-buffered formalin solution (CellStor, CellPath, Newtown Powys, UK) at room-temperature (RT) before being immersed in 30% sucrose solution until use, followed by embedding in cryogel for cryosectioning at 10 µm using a cryostat (MICROM International GmbH, part of Thermo Scientific CryoStar NX50, Walldorf, Germany). The sections were mounted onto SuperFrost Plus Adhesion microscope slides (Epredia^TM^, 22-042-941, Thermo Fischer Scientific, Waltham, MA, USA).

#### 4.4.2. Immunohistochemistry

The cryosections were air-dried for 30 min, washed (3 × 5 min) with phosphate-buffered saline (PBS, Gibco, Waltham, MA, USA), boiled in pH 6 citrate buffer for antigen retrieval (Agilent, S1699, Santa Clara, CA, USA), and washed in Tris-buffered saline with 0.1% Tween-20 (TBS-T, Sigma-Aldrich) for permeabilization. Afterwards, the cells were blocked with 20% preimmune goat serum (PIG, Thermo Fisher Scientific, 31872, Waltham, MA, USA) in Tris-NaCl-blocking buffer (TNB) for one hour at RT. Afterwards, the samples were incubated overnight at 4 °C with rabbit anti-cleaved caspase-3 (CC3) (1:200, Cell Signaling, 9661, Leiden, The Netherlands), and rat anti-KI-67 (Antigen Kiel 67) (1:1000, eBioscience™, 14-5698-82, Thermo Fischer Scientific, Waltham, MA, USA) diluted in 5% PIG in TNB. After being washed with TBS-T (3 × 5 min), the cells were incubated with secondary goat anti-rat Alexa Fluor 568 (Invitrogen^TM^, A-11077, diluted 1:200 in TNB, Thermo Fischer Scientific, Waltham, MA, USA), and goat anti-rabbit Alexa Fluor 647 Plus (Invitrogen^TM^, A32733, diluted 1:1000 in TNB, Thermo Fischer Scientific, Waltham, MA, USA). The cells were then washed with TBS-T followed by incubation (15 min) with 4′,6-diamidino-2-phenylindole (DAPI, 5 µg/mL in PBS; Sigma-Aldrich, D9542, Merck KGaA, Darmstadt, Germany). Subsequently, the cryosections were mounted with Molecular Probes^TM^ ProLong^TM^ Diamond Antifade Mountant with DAPI (Thermo Fischer Scientific, 15810083, Waltham, MA, USA) and stored at −20 °C. A Nikon Eclipse Ti-E inverted widefield microscope (Nikon Instruments, Amstelveen, The Netherlands) with a 20× objective connected to a Prime BSI sCMOS camera was used to visualize cells in the cardiac models that were positive for the distinct markers. ImageJ version 1.54 was used to analyze the number of cells positive for cleaved caspase-3 or Ki67 on the basis of intensity measurements with rolling ball correction via a nuclear mask on the basis of the DAPI signal.

### 4.5. Adenosine Triphosphate (ATP)-Level Quantification

The cardiac organoids were transferred to white opaque 96-well F-bottom plates (Greiner, 655083, Vilvoorde, Belgium) with standard culture medium added to the wells. One single organoid was transferred to a single well in the 96-well plate to maintain equal organoid concentration per condition. An equal volume of the CellTiter-Glo^®^ 3D reagent from the CellTiter-Glo^®^ 3D Cell Viability Assay (Promega, G9682, Leiden, The Netherlands) was added. The contents were vigorously mixed for 5 min on a plate shaker to induce cell lysis and incubated for an additional 25 min to stabilize the luminescent signal. Luminescence was recorded via a CLARIOstar Plus Microplate Reader (BMG LABTECH, Ortenberg, Germany).

### 4.6. Assessment of Functional Beating

Real-time videos of beating cardiac organoids were recorded as .AVI files via a Nikon Eclipse Ti-E inverted microscope (Nikon Instruments, The Netherlands) at 37 °C with a 5× objective (rate: 50 fps, duration: 30 s, no compression). The open-source software tool MUSCLEMOTION V1.0 [154] was used to quantify cardioid contraction profiles on the basis of various parameters including contraction duration, relaxation time, peak-to-peak time, and 10-to-10 transient time in milliseconds (ms). The means with standard deviations were included in the individual graphs for each individual parameter.

### 4.7. Bulk RNA Sequencing (RNA-Seq)

Total RNA was extracted from cardiac organoids using the Maxwell^®^ RSC simplyRNA Tissue Kit (Promega, AS1340, Leiden, The Netherlands) according to manufacturer’s protocol. Sample preparation for sequencing was performed by Novogene (Münich, Germany). Messenger RNA was purified from total RNA using poly-T oligo-attached magnetic beads. After fragmentation, the first strand cDNA was synthesized using random hexamer primers, followed by the second strand cDNA synthesis using dTTP, end repair, A-tailing, adapter ligation, size selection, amplification, and purification. Sequencing was performed using a Illumina NovaSeq X (Illumina Inc., San Diego, CA, USA). Preprocessing of the RNA-seq data was performed with nfcore/rnaseq version 3.25.0 [155,156] using default parameters, except fastp was used as trimmer and salmon was run with “--gcBias”. Genome and associated annotation files were obtained from ENSEMBL (GRCh38, release 115). Differential expression analysis was performed with nfcore/differential abundance version 1.5.0 [155,157] with the default options.

### 4.8. Statistical Analysis

Statistical analyses were performed in GraphPad Prism version 10.4.0 for Windows (GraphPad Software, San Diego, CA, USA, www.graphpad.com). The results represent the average of two-to-three independent experiments as biological replicates ± standard deviation (SD). For each endpoint, outliers were identified via Grubb’s test, which is based on a test statistic that is calculated from the most extreme data point, and corresponds to a *p* value that represents the likelihood of detecting the outlier. The data were first tested for normality and homoscedasticity. To test for significant group differences, the Kruskal–Wallis test was used as a nonparametric test when data was not normally distributed with unequal variances. *p* values were corrected for multiple comparisons via Dunn’s test. Statistical significance was assumed when *p* values were <0.05.

## 5. Conclusions

In conclusion, this study shows that RPM-simulated µg and accelerated ^252^Cf exposure have different detectable effects on human cardiac organoids under the conditions tested. Simulated µg produced limited changes in the selected phenotypic readouts, whereas ^252^Cf exposure altered several viability- and function-related measures. Ambient-temperature-preserved organoids retained measurable beating during recovery after irradiation, although preservation also induced transient changes and its apparent benefit cannot yet be assigned to a specific radioprotective mechanism. These proof-of-concept findings justify further validation of ambient, non-cryogenic preservation as a promising and practical option for selected cardiac organoid workflows, including studies where simplicity, scalability, and reduced logistical burden are critical. In the context of space biology, such approaches could facilitate more flexible experimental workflows and broaden the use of advanced 3D human tissue models in both ground-based analog studies and future missions to the ISS and beyond.

## Data Availability

The original contributions presented in this study are included in the article/Supplementary Materials. Further inquiries can be directed to the corresponding authors. The data that support the findings of this study are not openly available for reasons of sensitivity and are available from the corresponding author upon reasonable request. Data are located in controlled access data storage at the Belgian Nuclear Research Center (SCK CEN).

## Supplementary Materials

The following supporting information can be downloaded at https://www.mdpi.com/article/10.3390/ijms27198796/s1.
