# Supplementary material for: Ambient Temperature Preservation Supports Recovery of Beating Capacity in Human Cardiac Organoids After Exposure to Simulated Space Stressors
Source: Int J Mol Sci. 2026 Sep 30;27(19):8796. doi: 10.3390/ijms27198796 (PMC13665183; doi:10.3390/ijms27198796)
Supplement: Supplementary file 1 [file ijms-27-08796-s001.zip › ijms-4566451-supplementary.pdf]

Supplementary Materials

1. Results

1.1. Number of beating organoid

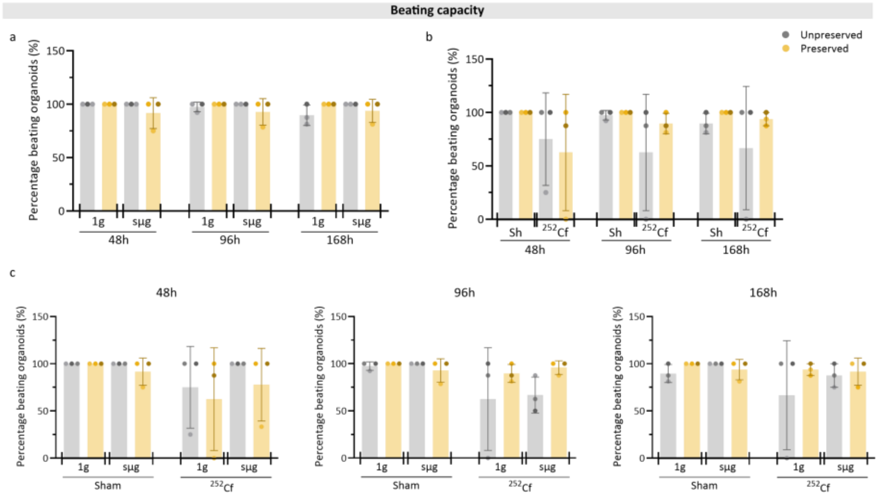

Figure S1: Visual observation of beating cardiac organoids. Data points represent averages of observed beating organoids (8-16 replicates). (**a**) Simulated microgravity (sµg) exposure slightly increased the number of beating organoids over time when unpreserved, while a reduction in beating organoids was observed in preserved organoids. (**b**) ^{252}Cf radiation reduced the number of beating organoids more when unpreserved. (**c**) Combined exposure affected number of beating organoids more when unpreserved. sµg: simulated microgravity; Sh: sham; ^{252}Cf: radiation.

1.2. ^{252}Cf radiation and simulated microgravity exposure of preserved and unpreserved cardiac organoids

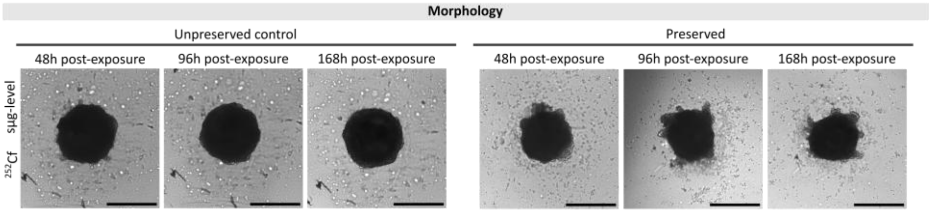

Figure S2: Brightfield images of unpreserved and preserved cardiac organoids post-exposure to ^{252}Cf radiation and simulated microgravity (sµg). Scale bar: 500 µm.

1.3. ^{60}Co radiation alters viability and beating function of cardiac organoids

Acute exposure with a Cobalt-60 (^{60}Co) γ-rays source was used to evaluate the effect of acute γ-rays exposure on the viability and function of preserved and unpreserved cardiac organoids. Size measurements based on brightfield images (Supplementary material - Figure S3a) showed a slightly reduced organoid diameters of irradiated unpreserved samples after 168 h (474 ± 99 μm) compared to sham controls (590 ± 101 μm) ($p < 0.01$), and compared to an earlier 48 h timepoint (601 ± 73 μm) ($p < 0.0001$) (Supplementary material - Figure S3b). This adverse radiation effect was also detected when evaluating ATP levels after 168 h post-exposure, where ATP levels of irradiated preserved organoid were 56% lower than sham controls ($p < 0.05$) (Supplementary material - Figure S3c). However, this effect was not detected in unpreserved cardiac organoids while exposed to ^{60}Co radiation. Beating capacity was altered by ^{60}Co radiation through reduced peak-to-peak time, indicating higher beating frequency, for unpreserved organoids at 48 h post-exposure, and for preserved organoids at 96 h post-exposure (Supplementary material - Figure S3g). Moreover, various beating parameters, including contraction duration, relaxation time, 10-to-10 transient time and peak-to-peak time, significantly increased over time for preserved irradiated organoids. Interestingly, no differences in beating capacity were observed when comparing preserved with unpreserved cardiac organoids upon exposure to ^{60}Co radiation.

Immunofluorescence images of cardioid cross sections revealed a 55% reduction in Ki67-positive cells ($p < 0.001$) and 59% increase in CC3-positive cells ($p < 0.05$) after 48 h post-exposure in unpreserved cardiac organoids compared to sham controls (Supplementary material - Figure S4a, S4b, S4c). This adverse radiation effect was also observed in preserved organoids with a 52% reduction after 168 h ($p < 0.05$), despite the 69% increase at 96 h, probably due to highly variated data ($p < 0.001$) (Supplementary material - Figure S4a, S4b). Increased apoptosis in preserved irradiated cardiac organoids compared to sham controls occurred after 48 h and 96 h post-exposure, with an increase of 60% and 70% respectively (Supplementary material - Figure S4a, S4c).

Overall, these results imply that size, ATP levels and proliferative capacity was reduced due to γ-rays irradiation, with a later proliferative effect when organoids were preserved. However, ATP levels were altered only in preserved organoids, without size reduction. Beating capacity was altered through higher beating frequency, which occurred earlier post-exposure for unpreserved organoids compared to preserved organoids. Furthermore, increased apoptosis was observed in unpreserved organoid after 48 h and 96 h, and only at this first timepoint for preserved cardiac organoids.

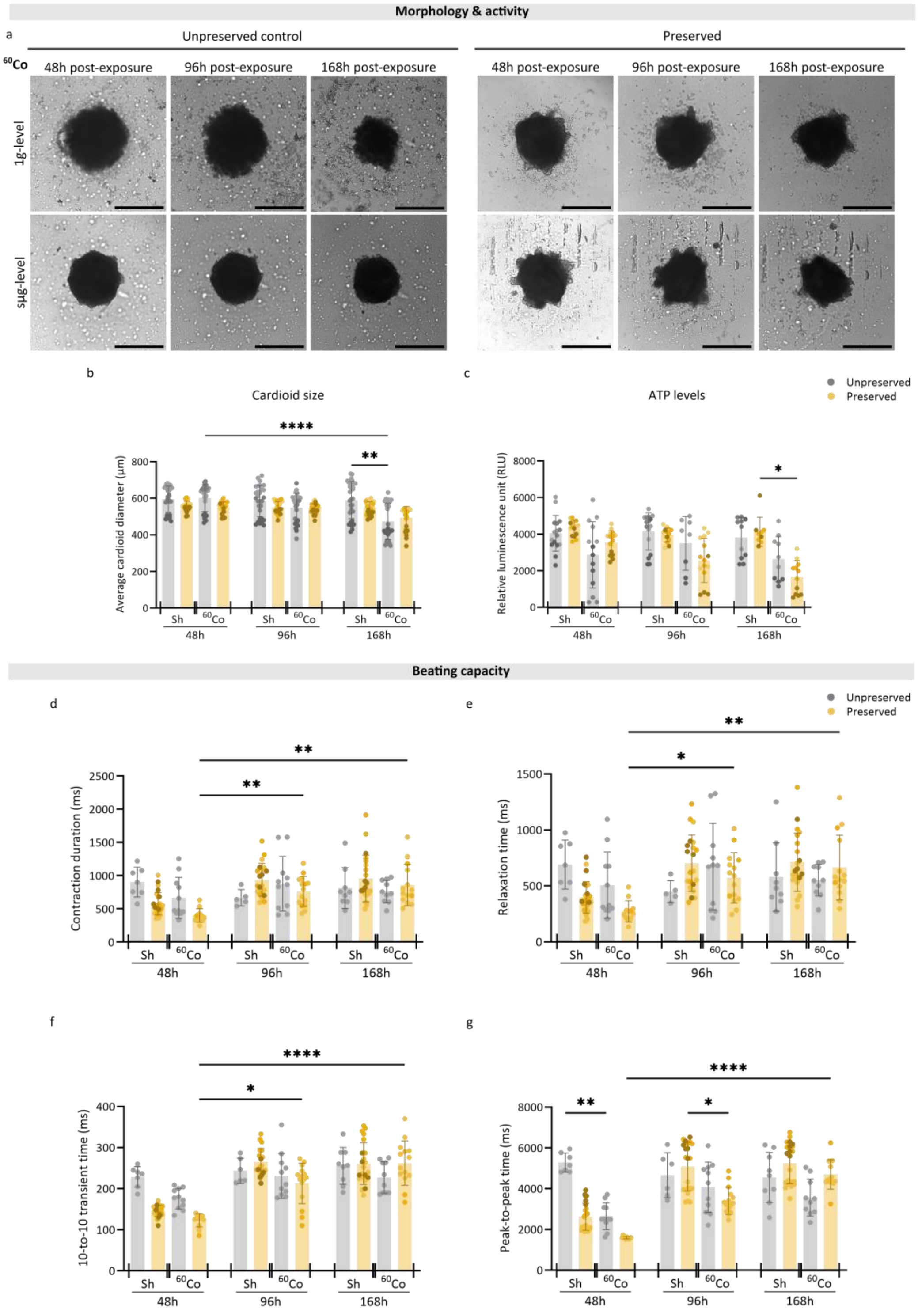

Figure S3: ^{60}Co radiation reduces size, ATP levels and beating capacity of cardiac organoids after 168 h post-exposure. (**a**) Brightfield images of unpreserved and preserved cardiac cardioids post-exposure to ^{60}Co radiation. Scale bar: 500 μm. (**b**) Average cardioid diameter of sham and irradiation cardiac organoids. (**c**) Averaged

cardioid ATP levels of sham and irradiated cardiac organoids. ATP: adenosine triphosphate. Beating parameters of cardiac organoids: contraction duration (**d**), relaxation time (**e**), 10-to-10 transient time (**f**) and peak-to-peak time (**g**) in milliseconds (ms). Shades represent biological replicates (N=3). The data are presented as the means ± SD. The Kruskal-Wallis test with Dunn's multiple comparisons test was used. * $p < 0.05$; ** $p < 0.01$; **** $p < 0.0001$.

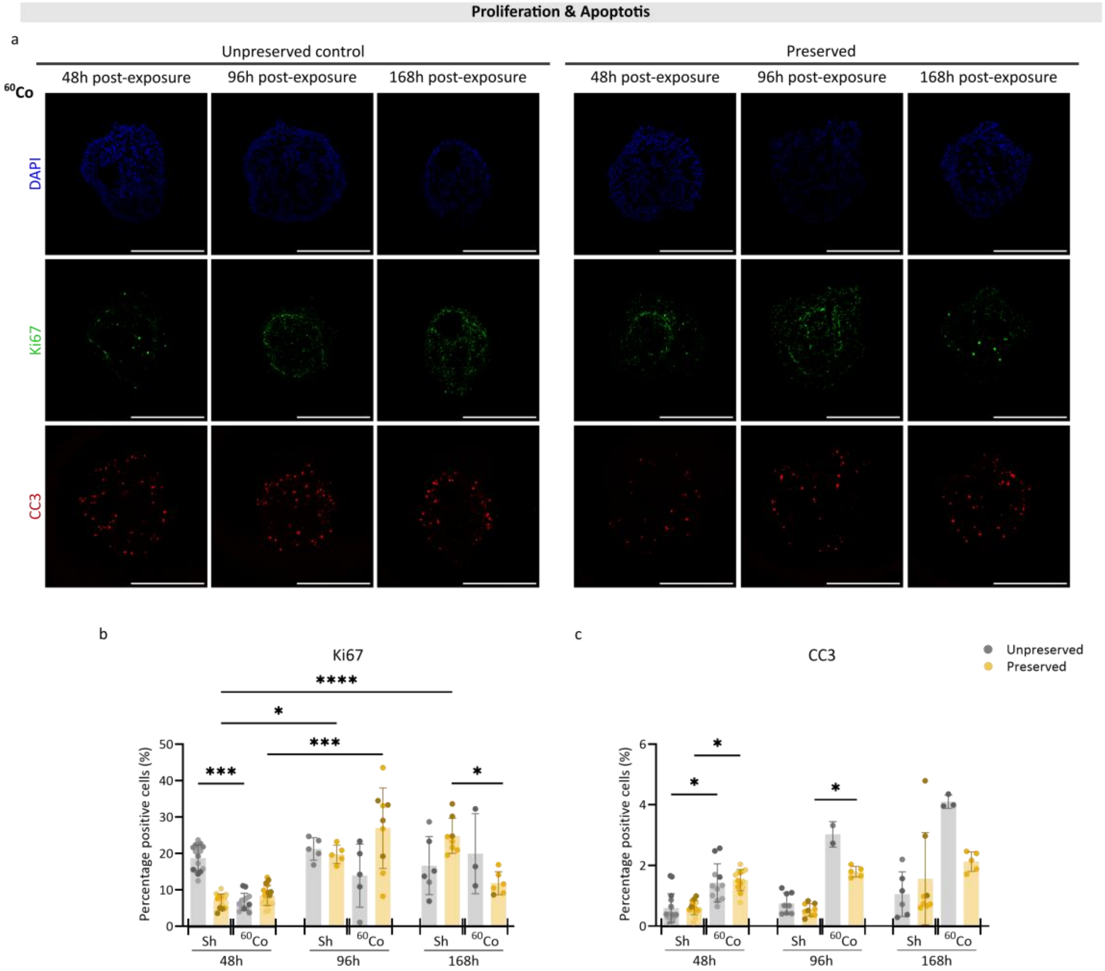

Figure S4: Exposure to ^{60}Co radiation initially decreases proliferation capacity and increases apoptosis in cardiac organoids. (**a**) Fluorescence images of cardiac organoid cross sections. Scale bar: 300 μm. DAPI: nuclear marker; Ki67: proliferation marker; CC3: apoptosis marker. (**b**) The percentage of Ki67-positive cells, which is indicative of the number of proliferative cells (N=3). (**c**) The percentage of CC3-positive cells, which is indicative of the number of apoptotic cells (N=3). The data are presented as mean ± SD, and the Kruskal-Wallis test with Dunn's multiple comparisons test was used. * $p < 0.05$; *** $p < 0.001$; **** $p < 0.0001$.

1.4. ^{60}Co radiation with simulated microgravity exposure affects ATP levels, proliferative capacity and induces apoptosis

Combined exposure of cardiac organoids to ^{60}Co radiation and simulated µg reduced ATP levels with 37% after 96 h post-exposure in unpreserved organoids compared to 1g-level sham controls ($p < 0.05$) (Supplementary material - Figure S5b), without any effect at other timepoints or based on size measurements (Supplementary material - Figure S5a). When comparing unpreserved exposed organoids to µg-level sham controls, a reduction in size after 96 h (11%) and 168 h (15%), and reduced ATP levels were observed at all timepoints (30% at 48 h; 42% at 96 h; 49% at 168 h), indicative of an adverse radiation effect at µg-level (Supplementary material - Figure S5a, S5b). Furthermore, combined exposure of preserved organoids did not affect their size or ATP levels (Supplementary material - Figure S5a, S5b). Evaluation of beating capacity upon combined exposure showed an increased contraction duration after 168 h post-exposure in preserved organoids compared to µg-level controls, indicative of an effect induced by simulated µg exposure when irradiated (Supplementary material - Figure S5k). This effect was also observed for the 10-to-10 transient time at 96 h and 168 h (Supplementary material - Figure S5i, S5m), and for the peak-to-peak time at 48 h (Supplementary material - Figure S5f), resulting in lower contraction frequency that are held longer in the contracted state for preserved organoids. Additionally, 10-to-10 transient time was reduced after 48 h (Supplementary material - Figure S5e) and peak-to-peak time was increased after 168 h (Supplementary material - Figure S5n) in unpreserved organoids that were exposed to ^{60}Co radiation and µg-level compared to 1g-level irradiated organoids, indicating a simulated µg-induced. Moreover, a radiation-induced effect was observed when comparing exposed unpreserved organoids to µg-level sham controls, with reduced 10-to-10 transient time after 48 h (Supplementary material - Figure S5e) and increased peak-to-peak time after 168 h (Supplementary material - Figure S5n). Interestingly, combined exposure of preserved organoids increased 10-to-10 transient time, so time in contracted state, after 168 h post-exposure (Supplementary material - Figure S5m). When comparing exposed preserved with unpreserved cardiac organoids, no significant differences were found. However, after 96 h post-exposure, no measurable beating was observed for unpreserved organoids when exposed to both stressors, while preserved organoids retained beating capacity (Supplementary material - Figure S5g, S5h, S5i, S5j).

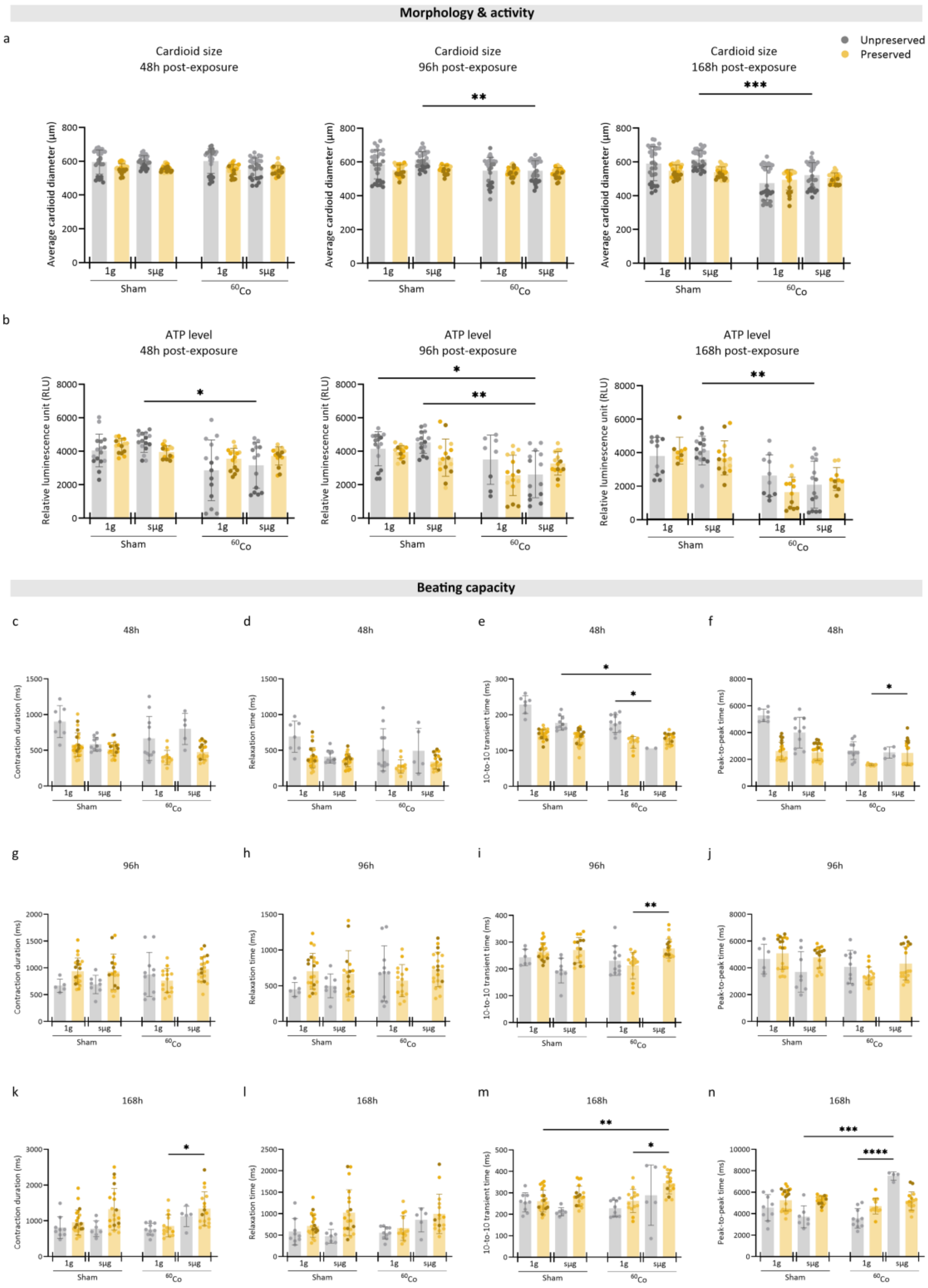

Figure S5: Combined exposure reduced ATP levels of unpreserved cardiac organoids at 96 h, while retaining beating capacity. (**a**) Average cardioid diameter of sham and irradiation cardiac organoids after 48 h, 96 h and 168 h post-exposure. (**b**) Averaged cardioid ATP levels of sham and irradiated cardiac organoids after 48 h, 96 h and 168

h post-exposure. ATP: adenosine triphosphate. Beating parameters of cardiac organoids after 48 h, 96 h and 168 h post-exposure: contraction duration (**c, g, k**), relaxation time (**d, h, l**), 10-to-10 transient time (**e, i, m**) and peak-to-peak time (**f, j, n**) in milliseconds (ms). Shades represent biological replicates (N=3). The data are presented as the means ± SD. The Kruskal-Wallis test with Dunn's multiple comparisons test was used. . * $p < 0.05$; ** $p < 0.01$; *** $p < 0.001$; **** $p < 0.0001$.

Immunohistochemistry of combined exposed cardiac organoids showed a significant decrease of 53% in Ki67-positive cells and increase of 67% in CC3-positive cells when unpreserved at 48 h post-exposure compared to sham 1g-level controls (Supplementary material - Figure S6a, S6b, S6c). This adverse combined exposure effect on proliferative capacity was detected in preserved organoids at a later timepoint after 168 h post-exposure ($p < 0.05$) (Supplementary material - Figure S6a, S6b). However, increased apoptosis was already observed after 48 h for this preserved condition ($p < 0.05$) (Supplementary material - Figure S6a, S6c), but stabilized over time to levels compared to controls. Moreover, ^{60}Co radiation added to μg-level exposure in unpreserved cardiac organoids reduced the number of Ki67-positive cells at 48 h post-exposure ($p < 0.05$), and increased the number of CC3-positive cells at all timepoint post-exposure (Supplementary material - Figure S6a, S6b, S6c). This effect is indicative of an adverse radiation effect when exposed to μg-levels.

Overall, combining ^{60}Co radiation with μg-level exposure reduced ATP levels, proliferative capacity and increased apoptosis in unpreserved cardiac organoids. These effects on proliferation and apoptosis were also observed in preserved cardiac organoids, without affecting size and ATP measurements. Beating capacity analysis only showed a single effect of combined exposure of preserved cardiac organoids based on 10-to-10 transient time, with overall retainment of beating function post-exposure for preserved organoids.

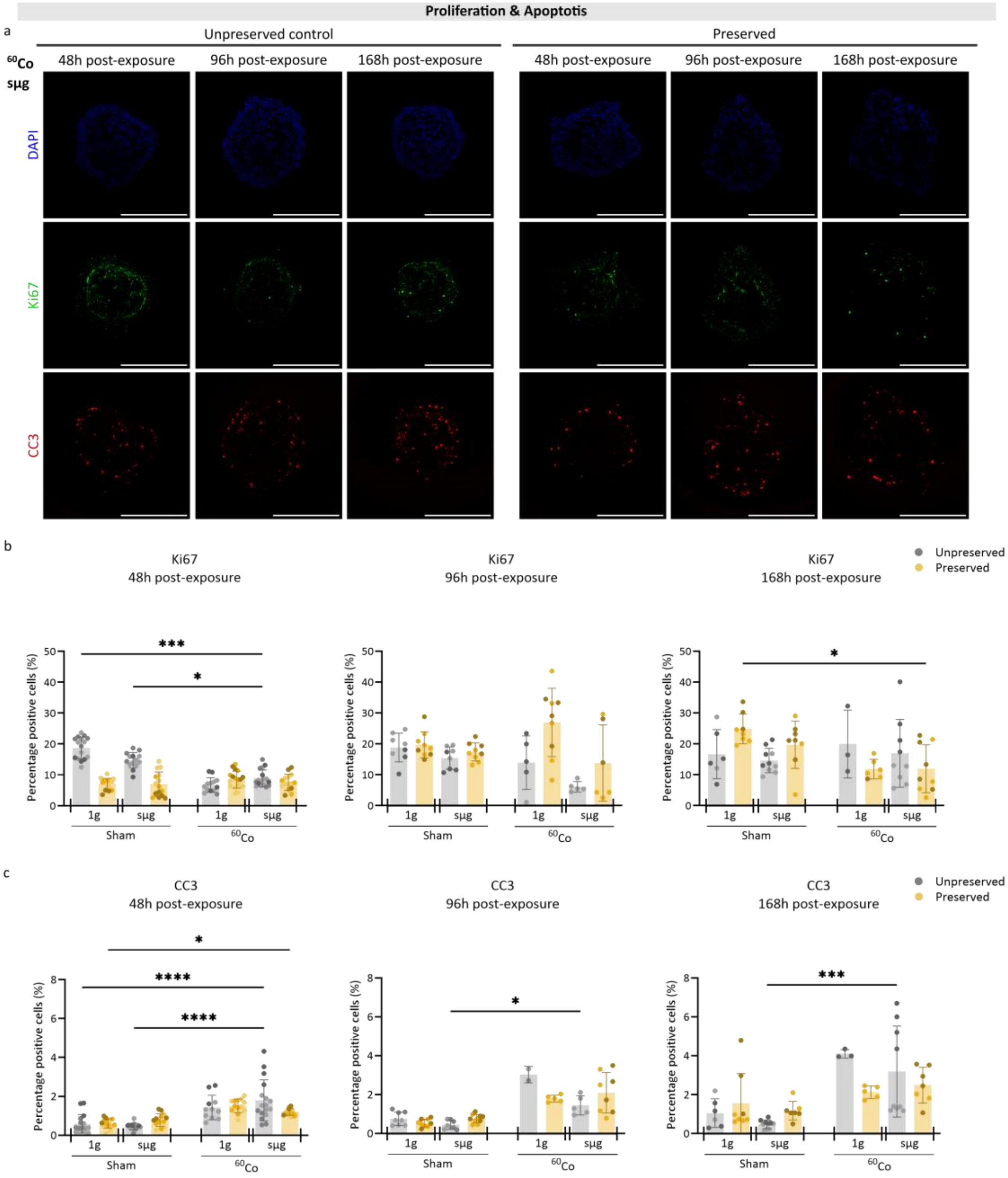

Figure S6: Combined exposure of space stressors reduced proliferation and increased apoptosis levels of cardiac organoids. (**a**) Fluorescence images of cardiac organoid cross sections. Scale bar: 300 µm. DAPI: nuclear marker; Ki67: proliferation marker; CC3: apoptosis marker. (**b**) The percentage of Ki67-positive cells, which is indicative of the number of proliferative cells (N=3). (**c**) The percentage of CC3-positive cells, which is indicative of the number of apoptotic cells (N=3). The data are presented as mean ± SD, and the Kruskal-Wallis test with Dunn's multiple comparisons test was used. * $p < 0.05$; *** $p < 0.001$. **** $p < 0.0001$.

1.5. Enrichment analysis of differentially expressed gene sets

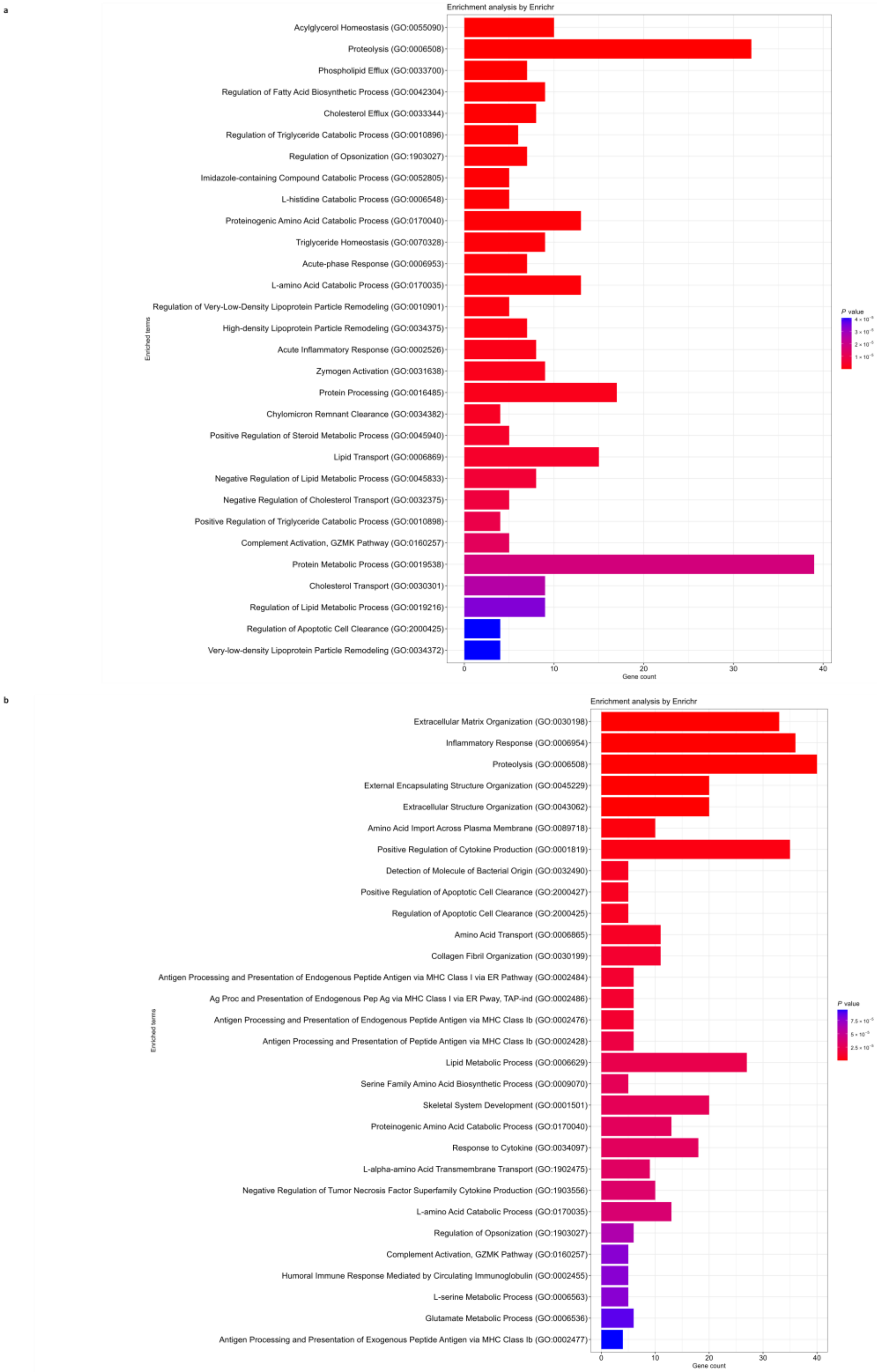

Figure S7: Enrichment analysis of differentially expressed downregulated gene sets upon exposure to simulated microgravity (sμg) (**a**) and ^{252}Cf radiation (**b**) while preserved at ambient temperature.

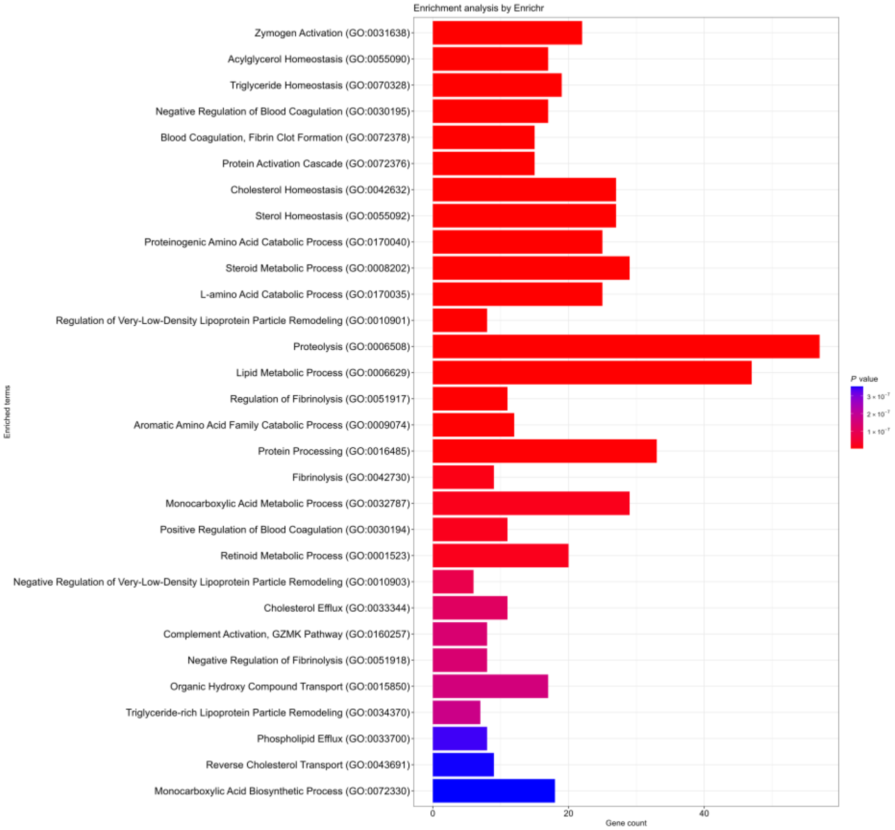

Figure S8: Enrichment analysis of differentially expressed downregulated gene sets upon combined exposure to simulated microgravity (sμg) and ^{252}Cf radiation while preserved at ambient temperature.
